# The mRNA MOXD1: Link to oxidative stress and prognostic significance in gastric cancer

**DOI:** 10.1515/med-2025-1271

**Published:** 2025-09-19

**Authors:** Youming Xiao, Xiqing Zhu, Cong Wang, Hongyu Gao, Zenghui Hao, Haibin Song, Zhaozhu Li

**Affiliations:** Department of Pediatric Surgery, The Second Affiliated Hospital of Harbin Medical University, Harbin, China; Department of Pediatric Surgery, The Sixth Hospital Affiliated of Harbin Medical University, Harbin, China; Department of Gastroenterological Surgery, Harbin Medical University Cancer Hospital, Harbin Medical University, Harbin, China

**Keywords:** gastric cancer, MOXD1, mRNA, oxidative stress, prognosis

## Abstract

**Purpose:**

Oxidative stress (OS) plays a key role in gastric cancer (GC). The purpose of this study was to investigate the role of the mRNA monooxygenase DBH-like 1 (*MOXD1*) in OS and evaluate its prognostic significance in GC.

**Methods:**

An OS risk score was constructed by unsupervised clustering analysis, the log-rank test, and least absolute shrinkage and selection operator–Cox analysis of OS-related genes. The Pearson correlation between *MOXD1* expression and the OS risk score was evaluated. Correlations between *MOXD1* expression and clinicopathological features in the training cohort were compared. CIBERSORT, ssGSEA, and ESTIMATE were used to analyze the effects of *MOXD1* on the immune microenvironment. Gene Ontology, Kyoto Encyclopedia of Genes and Genomes, and gene set enrichment analysis were used to elucidate the biological functions of the mRNAs. Immunohistochemistry for *MOXD1* was performed on patient tissue microarray (TMA) samples. Cox regression, log-rank tests, and chi-square analyses were used to investigate the clinicopathological features of the TMAs and associated *MOXD1* expression levels. A stable knockdown cell line was constructed in HGC-27 GC cells and investigated using cell counting kit-8 and Transwell assays.

**Results:**

The OS risk score was an independent prognostic factor for GC in the training cohort and was successfully combined with age and pTNM stage to construct a nomogram. *MOXD1* expression was positively correlated with the OS risk score and was highly expressed in patients with GC. *MOXD1* expression and the metastatic lymph node ratio in TMAs were found to be independent prognostic risk factors for GC. *MOXD1* knockdown inhibited the proliferation and invasion of HGC-27 cells.

**Conclusion:**

The mRNA *MOXD1* is a biomarker for both OS and GC. *MOXD1* expression can be used to evaluate GC prognosis and guide treatment.

## 1 Introduction

Gastric cancer (GC) is the fifth most common malignant tumor in the world and the fifth most common cause of cancer death [1]. Owing to the highly heterogeneous character of this malignant tumor, GC patients encounter great difficulties in obtaining a timely prognosis and receiving comprehensive treatment. With developments in sequencing technology, GC has been characterized in detail at the transcriptomic level, and the involvement of multiple biological pathways has been verified. In particular, oxidative stress (OS), lipid metabolism, and ferroptosis have been demonstrated to play important roles in GC pathobiology [2,3,4]. Moreover, new molecular targets for GC are constantly being identified, improving our understanding of GC etiology and pathogenesis and ultimately providing new guidance for treatment [5,6,7].

OS occurs when an imbalance between oxidants and antioxidants causes an increase in the level of reactive oxygen species (ROS). ROS are produced by mitochondria during high oxygen consumption, and they can destroy the structure of intracellular proteins and nucleic acids, thus disrupting cell homeostasis and potentially inducing tumorigenesis [8,9]. In thyroid tumors, Dogan et al. reported that OS was significantly greater at the tumor edge than at the tumor center (and also significantly greater than that in healthy thyroid tissue) [10], suggesting that tumorigenesis in thyroid cancer patients is related to an increase in OS. Moreover, OS is known to affect the proliferation, migration, and invasion of tumor cells, processes that are closely related to the occurrence and development of many malignant tumors, including GC, breast cancer, and bladder cancer [11,12,13]. On the basis of these considerations, Wang et al. developed a prognostic scoring system based on OS and ferroptosis that could better stratify patients with colorectal cancer [14]. OS is also known to influence the immune microenvironment of tumors. For example, high ROS levels are frequently accompanied by an increase in M2 macrophages and the inhibition of dendritic cells, thus promoting tumor progression [15,16]. Additionally, OS is associated with the proliferation of tumor-associated fibroblasts [17]. OS has also been shown to perturb numerous biological pathways, including the MAPK/JNK/ERK pathway and the transforming growth factor-β signaling pathway [17,18]. In conclusion, the role of OS in the occurrence and development of tumors is complex, and this role is worth exploring further and is likely to be richly targeted.

In Jee et al.’s animal experiments, MOXD1 was found to be highly correlated with monooxygenase and oxidoreductase activity. Further research is needed to determine whether MOXD1 regulates OS levels in the tumor microenvironment [19]. In the present study, the role of mRNA and protein levels of monooxygenase DBH-like 1 (*MOXD1*) in OS and GC was investigated. The gene for *MOXD1* is located on chromosome 6. MOXD1 is a member of the copper monooxygenase family and is known to modify proteins with copper ion binding and oxidoreductase activities [20]. In chronic obstructive pulmonary disease and diabetic kidney disease, *MOXD1* expression was found to be associated with disease occurrence, providing indirect evidence that *MOXD1* plays a role in chronic inflammation [21,22]. *MOXD1* is also a known senescence-related molecule, further confirming its relationship with immunity [23]. Additionally, *MOXD1* is a potentially important prognostic biomarker in bladder cancer, high-grade serous ovarian cancer (HGSOC), and hepatocellular carcinoma [24,25,26]. Shi et al. reported that *MOXD1* can bind to β3GnT2 and affect the protein glycosylation process. *MOXD1* knockdown induced endoplasmic reticulum (ER) stress, triggering the ER‒mitochondrial apoptosis pathway and modulating the progression of glioblastoma (GBM) [27]. Although *MOXD1* has been demonstrated to be a biomarker for early GC [28], its biological function and prognostic significance require further study.

## 2 Materials and methods

### 2.1 GC data

The GC data were obtained from 269 tumor patients who underwent radical gastrectomy at Harbin Medical University (HMU) Cancer Hospital. From this cohort (HMU-GC), tumor tissue samples, paratumorous normal tissue samples, and clinical data were collected and collated. The mRNA data from GSE15459 [29] supplementary (Table S2-4), GSE62254 [30] supplementary (Table S5), and The Cancer Genome Atlas (TCGA)-stomach adenocarcinoma (STAD) in Gene Expression Omnibus (GEO) and TCGA were additionally included in our analyses.

### 2.2 Data processing

First, the HMU-GC and TCGA-STAD data were converted into transcripts per kilobase million values. To increase the sample size, improve the power of statistical analysis, and improve the universality and reliability of research results, the ComBat algorithm [31] in the sva package was used to combine these two independent mRNA data into one training cohort (the HMU-TCGA cohort). This can eliminate systematic bias caused by different experimental conditions, sample processing or measuring equipment. Compared with other methods of integrating gene expression data, combat can provide robust batch effect correction in the case of small samples. The original CEL files for the GSE15459 and GSE62254 datasets were downloaded from GEO and independently merged into a validation cohort using the ComBat algorithm.

### 2.3 Structure of the OS risk score

Following the methods of Liu et al. [32], we first downloaded the “GOBP_RESPONSE_TO_OXIDATIVE_STRESS” gene set in MSigDB and intersected it with the training cohort to obtain 392 mRNAs (Additional file 1). Unsupervised clustering analysis using the ConsensusClusterPlus package in R programming language [33] showed that dividing the training queue into three groups has prognostic significance. OS-related genes (OSRGs) with prognostic significance were then analyzed in the C1 and C3 groups, and the tumor and paratumorous normal tissues were compared via Limma. Genes with |fold change| > 1.5 and a false discovery rate (FDR) < 0.05 were subsequently selected. Finally, a least absolute shrinkage and selection operator–Cox analysis based on the glmnet package was applied. The OS risk score was determined as follows:

\[{\mathrm{risk\; score}}=\mathop{\sum }\limits_{i=1}^{n}({\beta }_{i}{\mathrm{\times }}{{\mathrm{Exp}}}_{i}).]\]

A heat-map was used to provide a graphic illustration of gene expression and prognosis in the OS risk score. A log-rank test was used for the Kaplan‒Meier (K‒M) survival curve. Univariate and multivariate analyses of OS risk score, age, sex, and pTNM stage were performed (based on Cox regression). Independent prognostic factors in the training cohort were combined to construct a nomogram using the rms and survival packages. The performance of the nomogram was evaluated with a time-dependent receiver operating characteristic (timeROC) curve generated using the timeROC package, a calibration curve generated using the rms and survival packages, and a decision curve analysis (DCA) curve generated using the rmda package. The ggplot2 package was used for visualization.

### 2.4 Immune microenvironment analysis

The numbers of infiltrating immune cells in each tumor sample were estimated using ssGSEA and CIBERSORT. The immune score, stromal score, ESTIMATE score, and tumor purity were calculated using ESTIMATE. Tumor immune dysfunction and exclusion (TIDE) scores were calculated for patients using the TIDE database (http://tide.dfci.harvard.edu/). This surrogate biomarker was applied to evaluate patient suitability for immune checkpoint therapy (patients with a high TIDE score are not suitable for immunotherapy) [34]. The expression of *MOXD1* in different cell subsets of the immune microenvironment was explored using the GC public single-cell database (STAD-GSE134520 and STAD-GSE167297) on the website of the Tumor Immune Single-cell Hub (http://tisch.comp-genomics.org/).

### 2.5 Bioinformatics analyses

For the Gene Ontology (GO) and Kyoto Encyclopedia of Genes and Genomes (KEGG) analysis, the clusterProfiler package was used. ID conversion was performed using the org.Hs.eg.db package. The clusterProfiler package was also used in the gene set enrichment analysis (GSEA) to explore potential biological pathways. The reference genome was the hallmark gene set, and the selection conditions were as follows: |normalized enrichment score| > 1; nominal *P* value < 0.05; and FDR *Q* value < 0.25. The protein‒protein interaction (PPI) network was generated using STRING (version 12.5) (https://cn.string-db.org/). Somatic mutation data from the TCGA-STAD dataset were analyzed by the GDCquery_Maf() function (pipelines = “mutect2”) in the biolinks package, and the maftools package was used for identification and visualization [35,36].

### 2.6 Drug analysis

On the basis of the Profiling Relative Inhibition Simultaneously in Mixtures public drug susceptibility database [37], the area under the receiver operating characteristic curve (AUC) of each sample was estimated by ridge regression, and the chemotherapy response was predicted using the pRRophetic package. The prediction accuracy was evaluated by 10× cross-validation using the training cohort. Patients with a low AUC value show greater sensitivity to treatment.

### 2.7 Immunohistochemistry

The tissue microarrays (TMAs) were dewaxed, dehydrated using a gradient series, and rinsed with 3× concentrated phosphate-buffered solution (PBS) (5 min each) (DW0300, Dowobio, Shanghai, China), after which the antigens were retrieved in sodium citrate buffer (pH = 6) for 3 min at 120°C (DW2215, Dowobio, Shanghai, China). Finally, the TMAs were rinsed with 3× concentrated PBS (5 min each time), incubated with 3% H_2_O_2_ for 30 min (MM0750-500ML, MKbio, Shanghai, China), and then processed by immunostaining. First, the sections were blocked in goat serum for 1 h (Boster, USA). Next, a diluted anti-MOXD1 antibody (Bioss, bs-17733R; 1:150) was added, and the TMAs were then incubated overnight at 4°C. After the sections were rinsed with 3× concentrated PBS (5 min each time), goat anti-rabbit IgG (BA1039, Boster, USA) was added dropwise, and the TMAs were incubated at 37°C for 40 min. The color reaction was then developed using diaminobenzidine (AR1000, Boster, USA) staining. Finally, the sections were counterstained with hematoxylin (for nuclear staining) (MM1010-500ML, MKbio, Shanghai, China) and then viewed under a microscope. All the samples were examined by two pathologists, who evaluated the degree of positive cell staining (using a dividing line of 50%).

Immunohistochemical staining (IHC) is a commonly used method to evaluate the expression level of human epidermal growth factor receptor 2 (HER2): IHC 0: no staining or ≤10% of invasive cancer cells showed incomplete and weak cell membrane staining; IHC 1+: >10% of invasive cancer cells showed incomplete and weak cell membrane staining; IHC 2+: >10% of invasive cancer cells showed weak to moderate intensity of complete cell membrane staining or <10% of invasive cancer cells showed strong and complete cell membrane staining; IHC 3+: >10% of invasive cancer cells showed strong, complete, and uniform cell membrane staining. HER2 threshold is defined as: IHC score of 0 or 1+ is negative, IHC score of 2+, and further fish test is usually required to evaluate HER2 status by detecting the amplification of the HER2 gene. her2/cep17 ratio less than 2.0 or HER2 copy number less than 4 is negative, and her2/cep17 ratio greater than 2.0 or HER2 copy number greater than 6 is positive.

### 2.8 Cell line and transfection

GC HGC-27 cells were obtained from the Procell Life Science & Technology Co., Ltd. (Wuhan, China). The cells were cultured in a humidified incubator at 37°C (with 5% CO_2_) with RPMI-1640 supplemented with 20% fetal bovine serum and 1% penicillin/streptomycin solution (Procell Life Science & Technology Co., Ltd., Wuhan, China). To obtain a stable *MOXD1*-knockdown cell line, HGC-27 cells were infected with *MOXD1*-interfering and control viruses (OBiO Technology Corp., Ltd., Shanghai, China; https://www.obiosh.com/) at a concentration of 15 µg/mL supplementary (Table S1). The cells were incubated with the infection mixture for 24 h, after which the infection mixture was exchanged for fresh medium. To isolate stably transfected cells, the infected cells were screened on a gradient of puromycin (Dalian Bergolin Biotechnology Co., Ltd., Dalian, China), and this procedure was continued until no cell death occurred. The resulting stable cell lines were designated HGC-NC, HGC-MOXD1sh1, and HGC-MOXD1sh2.

### 2.9 RNA extraction and quantitative real-time PCR (qPCR)

TRIzol (Invitrogen, USA) was used to extract total RNA from the HGC-NC, HGC-MOXD1sh1, and HGC-MOXD1sh2 cell lines. The PrimeScript RT Reagent Kit (TaKaRa, China) was used for reverse transcription of each total RNA sample to obtain cDNA. qPCR was performed on a LightCyler 96 Roche system using SYBR PreMix Ex Tap II (TaKaRa, China) according to the manufacturer’s instructions. The results were analyzed using the 2^−ΔΔCt^ method, with *GAPDH* used as an internal reference. The primer sequences for both *MOXD1* and *GAPDH* are included in Additional file 1.

### 2.10 Western blot analysis

HGC-NC, HGC-MOXD1sh1, and HGC-MOXD1sh2 cells were lysed on ice for 30 min in RIPA buffer (Beyotime Biotechnology, Shanghai, PR China) containing phosphatase and protein inhibitors. The total protein concentrations of the resulting samples were quantified via a biochemical acid protein detection kit (Beyotime Biotechnology, Shanghai, China). After the protein samples were boiled for 10 min, 20 µg of each protein sample was loaded onto a 12% gel for separation by sodium dodecyl sulfate‒polyacrylamide gel electrophoresis. Next, the resolved proteins were transferred to a 0.22 µm polyvinylidene fluoride (PVDF) membrane (Millipore, USA). After transfer, the PVDF membrane was blocked with 5% skim milk powder for 2 h and then incubated overnight with primary antibody (at 4°C). The primary antibodies used were anti-MOXD1 (Bioss, bs-17733R; 1:1,000) and anti-GAPDH (Abcam, ab8245; 1:5,000). The membrane was then washed and incubated with a diluted anti-rabbit IgG (H + L) secondary antibody (ProteinTech, SA00001-2; 1:5,000) for 1 h. After further washing, the PVDF membrane was developed with enhanced chemiluminescence (ECL) reagent (Meilunbio, Dalian, China).

### 2.11 Cell proliferation measurement

A cell counting kit-8 (CCK-8) (Dalian Bergolin Biotechnology Co., Ltd., Dalian, China) was used to determine cell proliferation. HGC-NC, HGC-MOXD1sh1, and HGC-MOXD1sh2 cell lines were inoculated into 96-well plates (3 × 10^3^ cells per well). After adherence, a 10% CCK-8 mixture was added to the cells, and the plates were incubated for 45 min (away from direct light). Finally, fluorescence detection was performed at 450 nm on a miniature flat plate reader (at low speed). This experiment was repeated at 24, 48, and 72 h.

#### 2.11.1 Cell migration assay

HGC-NC, HGC-MOXD1sh1, and HGC-MOXD1sh2 cell layers were trypsinized, washed with 3× concentrated PBS, and then mixed with serum-free medium. Next, 200 µL of each cell suspension (8 × 10^4^ cells) was transferred to the upper chamber of the Transwell inserts, and 800 µL of complete culture medium was added to the lower chamber. After incubation for 24 h, the migrated cells were stained with 0.5% crystal violet for 30 min. The dishes were then wiped clean and sealed for subsequent observation.

### 2.12 Statistical analyses and bioinformatics analyses

Continuous variable data were analyzed via the Mann‒Whitney *U* test or Wilcoxon signed rank sum test. Chi-square analysis was used to analyze the correlations between clinicopathological features. Correlations between two continuous variables were measured via the Pearson test. The hazard ratio (HR) and 95% confidence interval (CI) were calculated using a Cox regression model. The K‒M survival curve was drawn by the log-rank test. For all the statistical analyses, a two-tailed *P* value of <0.05 was used to assess statistical significance. R statistical software (v4.0.2) was used. For GSEA, visualize the enrichment analysis results using the ggplot2 package. Immune infiltration algorithm: based on the CIBERSORT (CIBERSORT; R script analysis) core algorithm, utilizing the CIBERSORT Tx website (https://cibersortx.stanford.edu/), calculate the markers of 22 immune cells provided. Heatmap: Visualize heatmaps using the ComplexHeatmap package. The Wilcoxon rank sum test was used for differential analysis.


**Informed consent:** All study participants or their legal guardians provided signed informed consent forms.
**Ethical approval:** All procedures followed were performed according to the ethical standards of the Human Subjects Responsibility Committee (institutions and countries), as well as the 1964 Helsinki Declaration and subsequent editions (ethics approval number: KY2021-09).

## 3 Results

### 3.1 The prognostic significance of OS in patients with GC

**Figure 1:**
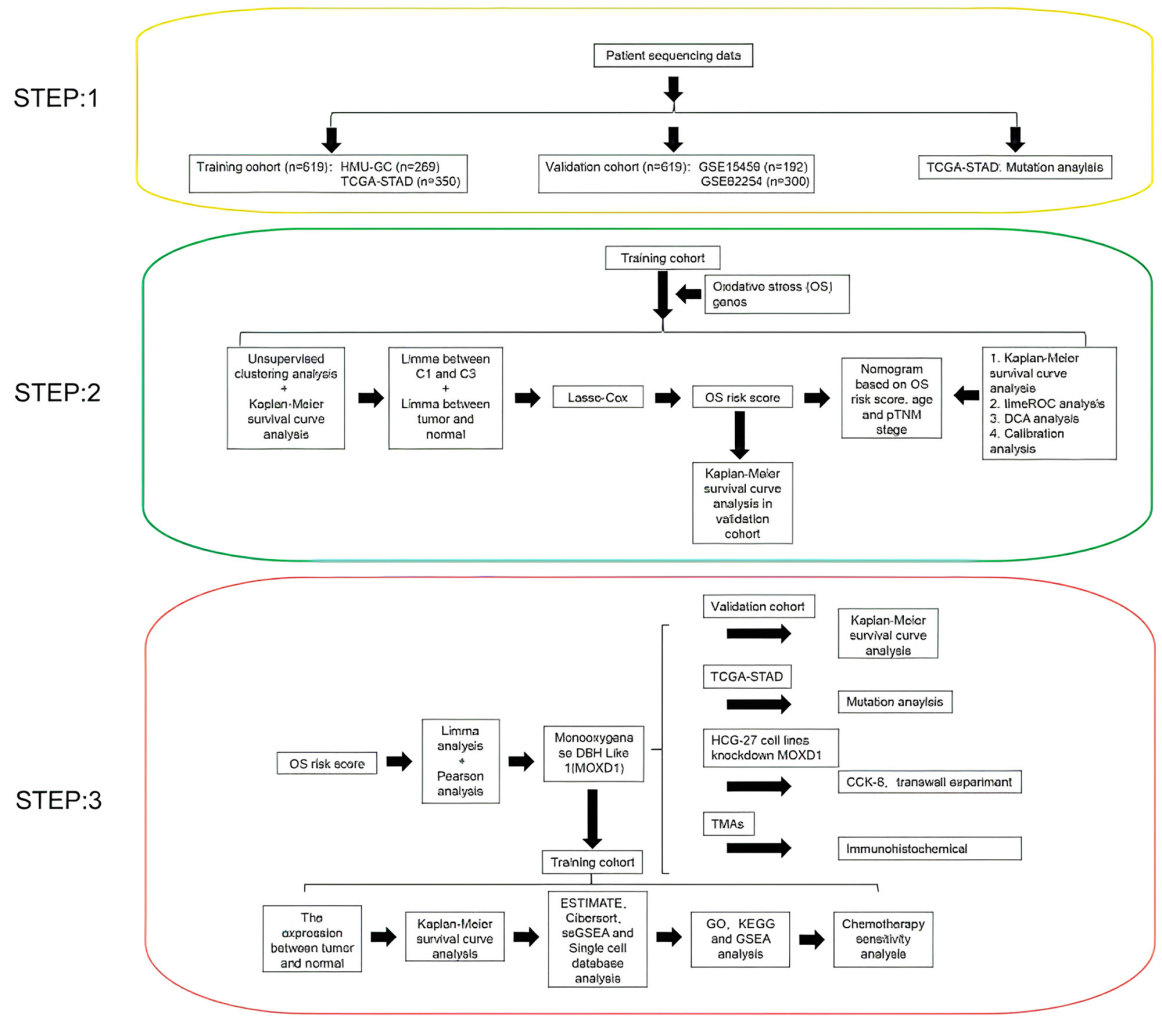
Flow chart of the research.

**Figure 2:**
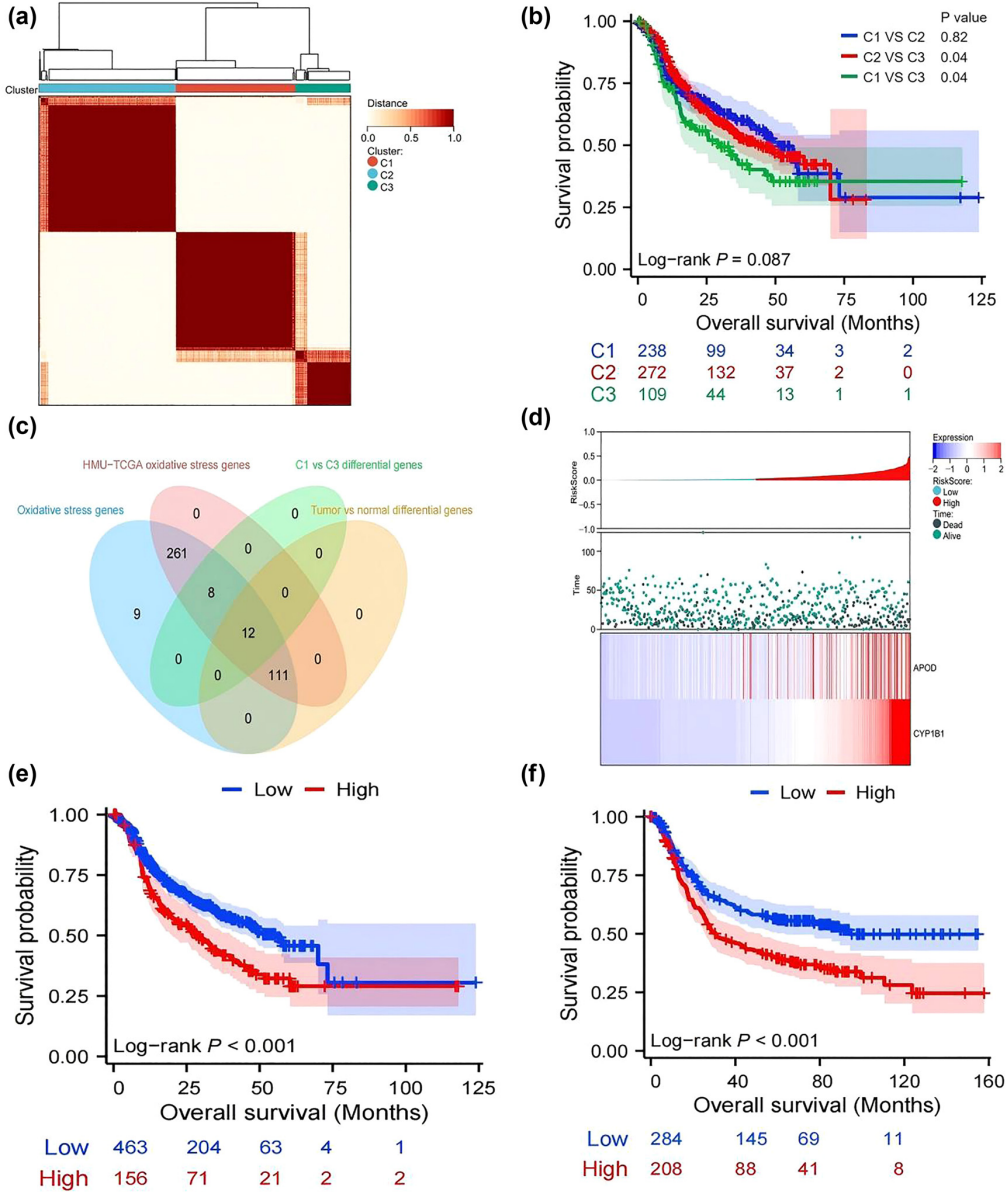
(a) Consensus heatmap of the three groups in the training cohort. (b) K‒M survival curves of the three groups in the training cohort. (c) Venn diagram showing the intersection of the oxidative stress (OS) gene set. (d) Heatmap of APOD and CYP1B1 mRNA expression and patient prognosis. (e) K‒M survival curve of the OS risk score in the training cohort. (f) K‒M survival curve of the OS risk score in the validation cohort.

The course of this study is illustrated in the flowchart shown in Figure 1. The training cohort was split into three groups (C1, C2, and C3) on the basis of the results obtained from unsupervised clustering analysis and survival analysis (Figure 2a and b; Figure S1a–c). As demonstrated by the survival curve (Figure 2b), the median survival times of the C1 and C3 groups were significantly different (51.1 months vs. 29.7 months). The differentially expressed genes (DEGs) obtained from comparisons of tumor and paratumorous normal tissue samples from the C1 and C3 groups were then identified (Figure 2c). In total, 12 OSRGs were identified using this method. After LASSO Cox regression analysis, two key genes, *APOD* and *CYP1B1*, were identified (Figure 2d; Figure S1d and e). The OS risk score was calculated to be 2.64243451531501 \[{e}^{-05}]\] *APOD + 0.00237358944377552*CYP1B1. All patients with high expression of *APOD* and *CYP1B1* (in both the training and validation cohorts) had a poor prognosis (all *P* < 0.05) (Figure S2a–d).

On the basis of their OS risk score, patients in the training cohort were organized into either a high OS risk score group or a low OS risk score group. As shown by the survival curve, the survival times of patients in the low-OS risk score group were significantly longer (HR, 1.565; 95% CI, 1.185–2.066; *P* < 0.001) (Figure 2e). A similar result was obtained with the validation cohort (HR, 1.626; 95% CI, 1.262–2.095; *P* < 0.001) (Figure 2f).

**Figure 3:**
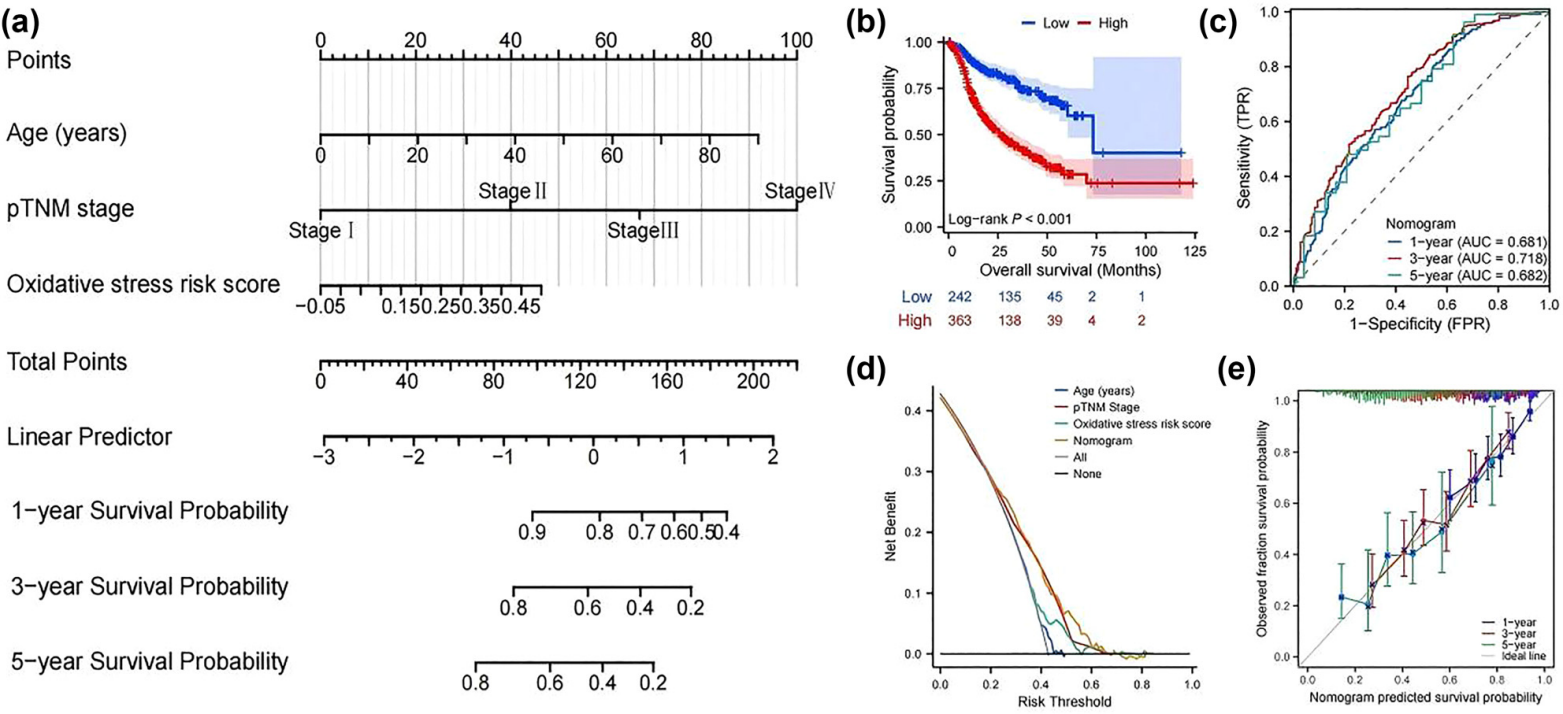
(a) Nomograms constructed by age, OS risk score, and pTNM stage in the training cohort. (b) K‒M survival curve for the nomogram. (c) timeROC curve for the nomogram. (d) DCA curve for the nomogram. (e) Calibration curve for the nomogram.

Next, Cox regression analysis of sex, age, pTNM stage, and OS risk score was performed on data from the training cohort. Our analysis revealed that age, pTNM stage, and the OS risk score were independent prognostic factors in patients (*P* < 0.001, *P* < 0.001, and *P* = 0.003, respectively) (Table 1). In the validation cohort, pTNM stage and OS risk score were again identified as independent prognostic factors in patients (*P* < 0.001 and *P* = 0.002). A nomogram was subsequently constructed using data from the training cohort (Figure 3a). The survival curve of the nomogram revealed that patients in the high-risk group had worse survival (HR, 2.977; 95% CI, 2.327–3.808; *P* < 0.001) (Figure 3b). The areas under the curves for evaluating postoperative survival at 1, 3, and 5 years were 0.681 (0.631–0.730), 0.718 (0.668–0.768), and 0.682 (0.561–0.824), respectively (Figure 3c). DCA, calibration curve analysis, and a C-index of 0.673 (0.655–0.690) provided further evidence of the good clinical application potential of the nomogram (Figure 3d and e).

**Table 1:** Univariate and multivariate analyses based on Cox regression in the training cohort

Characteristics	Total (*N*)	Univariate analysis		Multivariate analysis	
		Hazard ratio (95% CI)	*P* value	Hazard ratio (95% CI)	*P* value
**Age (years)**	619	1.019 (1.009–1.029)	**<0.001**	1.025 (1.014–1.036)	**<0.001**
**Sex**	619				
Male	398	Reference			
Female	221	0.891 (0.690–1.149)	0.373		
**pTNM stage**	605				
Stage I	83	Reference		Reference	
Stage II	151	2.782 (1.436–5.391)	**0.002**	2.625 (1.354–5.090)	**0.004**
Stage III	327	5.119 (2.781–9.422)	**<0.001**	5.043 (2.735–9.296)	**<0.001**
Stage IV	44	9.519 (4.746–19.089)	**<0.001**	11.170 (5.552–22.471)	**<0.001**
**Oxidative stress risk score**	619	8.520 (2.568–28.273)	**<0.001**	7.621 (2.010–28.901)	**0.003**

The pTNM stage is classified according to the AJCC 8th edition of the American Joint Committee on Cancer Staging Manual. Bold values represent the category headings.

### 3.2 Relationship between mRNA *MOXD1* expression and the OS risk score and its clinical significance

**Figure 4:**
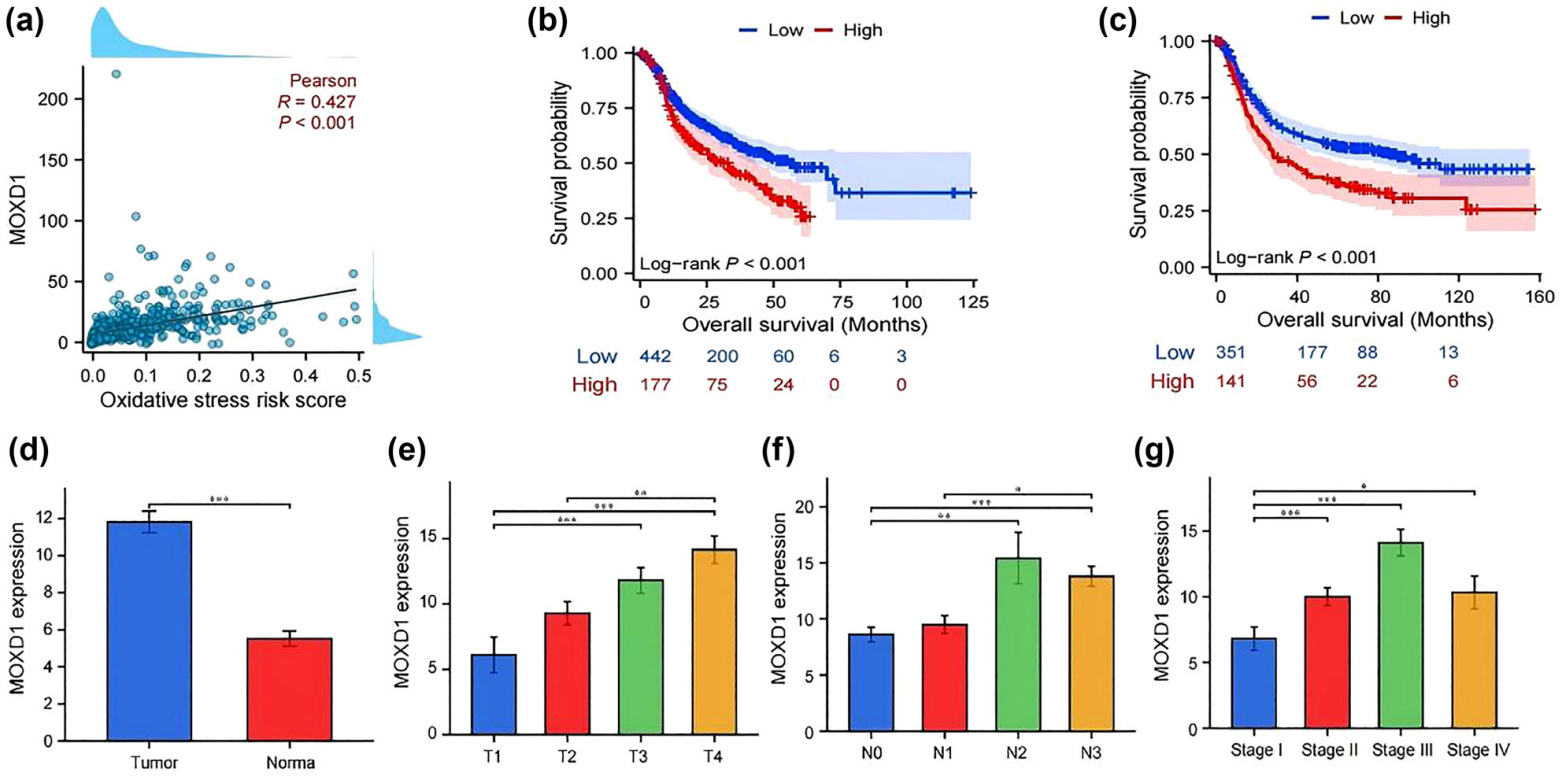
(a) Pearson correlation between *MOXD1* expression and the OS risk score in the training cohort. (b) K‒M survival curve for *MOXD1* expression in the training cohort. (c) K‒M survival curve for *MOXD1* expression in the validation cohort. (d) Differential expression of *MOXD1* between tumor and paratumorous normal tissue. (e–g) Differential expression of *MOXD1* in the T, N, and pTNM stages. *P < 0.05, **P < 0.01, ***P < 0.001.

*MOXD1* was present among the DEGs between the high- and low-OS risk score groups and was positively correlated with the OS risk score (*R* = 0.427, *P* < 0.001) (Figure 4a) (Additional file 1) (Figure S3a and b). In the training and validation cohorts, patients with high *MOXD1* mRNA expression all had poor survival (HR, 1.525; 95% CI, 1.166–1.996; *P* < 0.001) (HR, 1.591; 95% CI, 1.198–2.113; *P* < 0.001) (Figure 4b and c). In the training cohort, the mRNA expression level of *MOXD1* was independent of age and sex (Figure S4a and b). The mRNA expression of *MOXD1* in tumor tissue was significantly greater than that in adjacent normal tissue (Figure 4d). The mRNA expression of *MOXD1* in T3 and T4 patients was significantly greater than that in T1 and T2 patients (Figure 4e), the mRNA expression of *MOXD1* in N2 and N3 patients was significantly higher than that in N0 and N1 (Figure 4f), and the mRNA expression of *MOXD1* in stage III patients was the highest (Figure 4g), which indicated that *MOXD1* was closely related to the progression of GC. High mRNA expression of *MOXD1* in N0 and N1-2, stage II and III patients was associated with poor prognosis (all *P* < 0.05) (Figure S4c–k) (Table 2).

**Table 2:** Univariate and multivariate analyses based on Cox regression in the validation cohort

Characteristics	Total (*N*)	Univariate analysis		Multivariate analysis	
		Hazard ratio (95% CI)	*P* value	Hazard ratio (95% CI)	*P* value
**Age (years)**	492	1.007 (0.996–1.018)	0.230		
**Sex**	492				
Male	324	Reference			
Female	168	0.947 (0.726–1.234)	0.686		
**pTNM stage**	492				
Stage I	61	Reference		Reference	
Stage II	126	2.147 (1.041–4.427)	**0.038**	2.275 (1.101–4.697)	**0.026**
Stage III	168	5.257 (2.649–10.433)	**<0.001**	5.206 (2.622–10.335)	**<0.001**
Stage IV	137	12.832 (6.470–25.452)	**<0.001**	12.746 (6.419–25.307)	**<0.001**
**Oxidative stress risk score**	492	1.103 (1.056–1.153)	**<0.001**	1.074 (1.025–1.124)	**0.002**

The pTNM stage is classified according to the AJCC 8th edition of the American Joint Committee on Cancer Staging Manual. Bold values represent the category headings.

### 3.3 *MOXD1* and the GC tumor immune microenvironment

**Figure 5:**
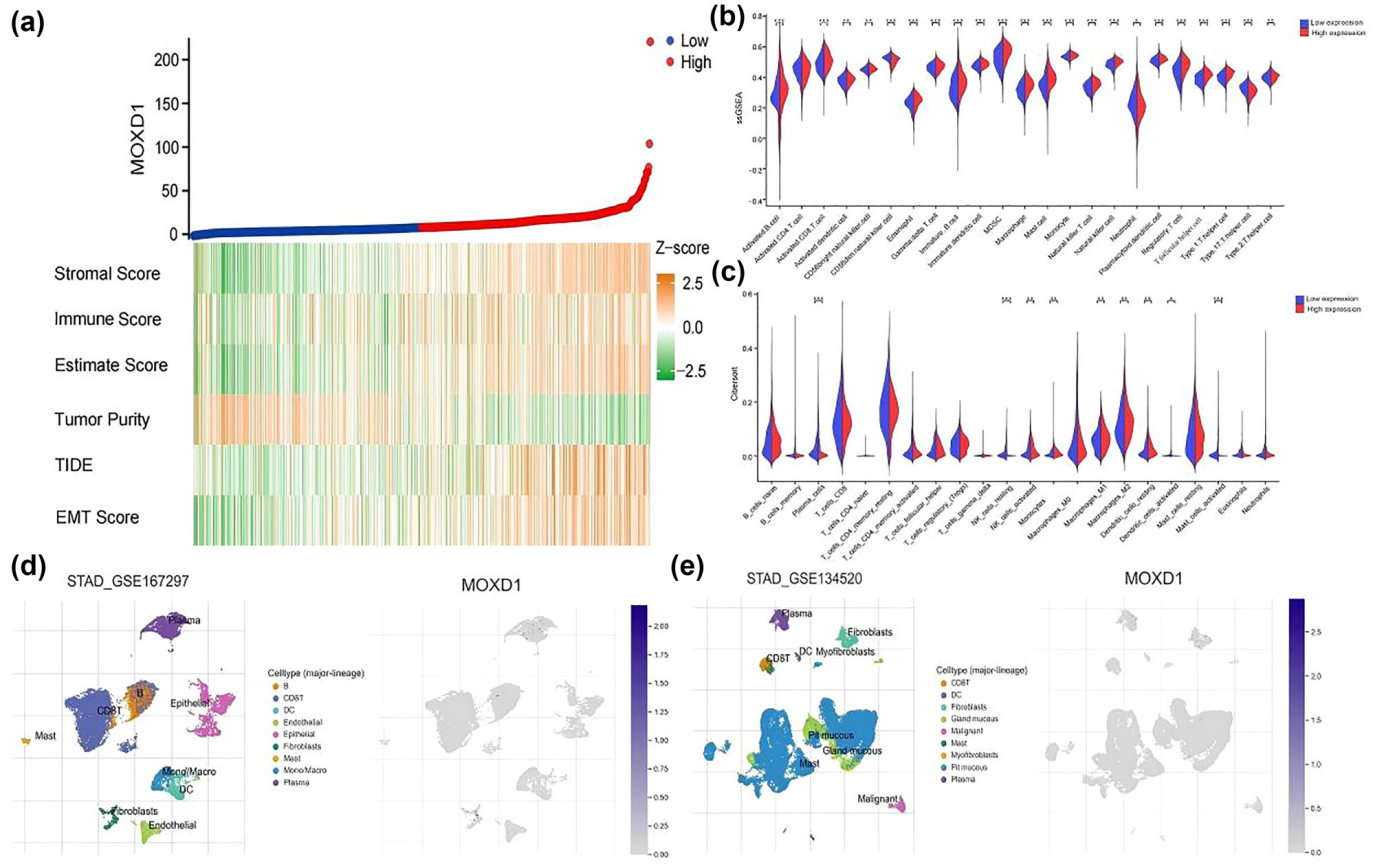
(a) Heatmap showing the correlations between *MOXD1* expression and the ESTIMATE score, stromal score, immune score, tumor purity, TIDE, and EMT score. (b) Immune infiltration in the high- and low-*MOXD1* expression groups in the training cohort (using the ssGSEA algorithm). (c) Immune infiltration in the high- and low-*MOXD1* expression groups in the training cohort (using the CIBERSORT algorithm). (d) and (e) Location of *MOXD1* expression in the STAD-GSE167297 and STAD-GSE134520 datasets.

Analysis was subsequently conducted on the basis of the median *MOXD1* expression level. Patients with high *MOXD1* expression presented higher ESTIMATE, stromal, and immune scores. Therefore, high expression of *MOXD1* is often accompanied by a strong immune response. Patients with high *MOXD1* mRNA expression also presented lower tumor purity and higher TIDE scores. Hence, patients with low *MOXD1* mRNA expression might be a potential population for immunotherapy. Additionally, high *MOXD1* mRNA expression was associated with a high epithelial–mesenchymal transition (EMT) score, providing further evidence that *MOXD1* is involved in the progression of GC (Figure 5a). According to the CIBERSORT scores, high mRNA expression of *MOXD1* was also associated with high tissue infiltration by M2 macrophages, activated NK cells, and resting dendritic cells. Conversely, low mRNA expression of *MOXD1* was associated with tissue infiltration by plasma cells, activated mast cells, and resting NK cells (Figure 5c). Our ssGSEA also revealed that *MOXD1* mRNA expression was largely related to immune cell type (Figure 5b). According to the single-cell database, *MOXD1* mRNA expression was especially associated with plasma cells and fibroblasts (Figure 5d and e).

### 3.4 Biological analysis of *MOXD1*

**Figure 6:**
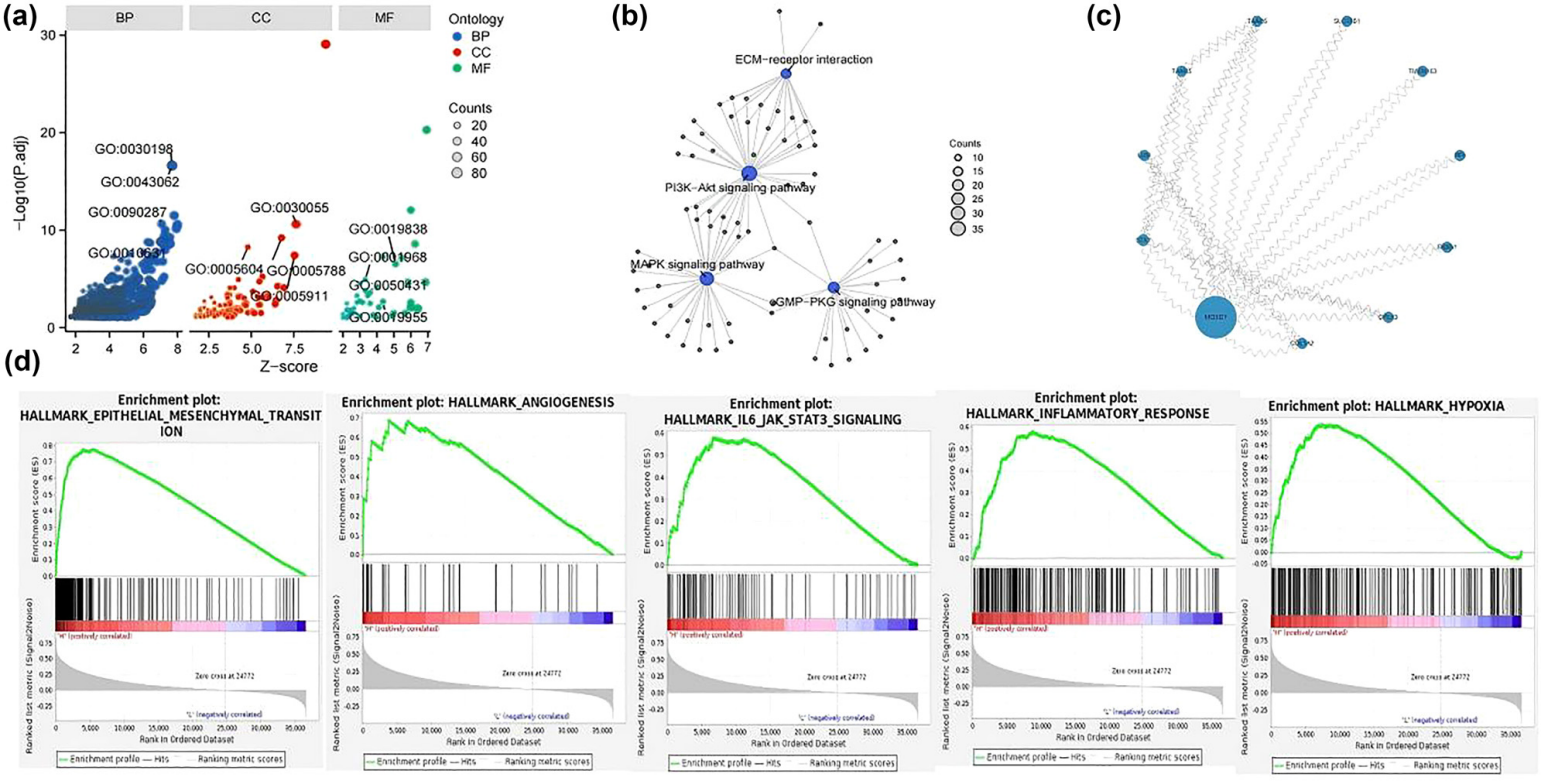
(a) GO analysis of *MOXD1*. (b) KEGG analysis of *MOXD1*. (c) PPI network analysis of MOXD1. (d) GSEA of *MOXD1*.

A DEG analysis was performed on the median *MOXD1* expression level via Limma, which yielded 6749 mRNAs (Figure S3c and d) (Additional file 1). GO analysis of biological process (BP) terms revealed that the mRNA *MOXD1* is involved mainly in extracellular matrix (ECM) organization, extracellular structure organization, and epithelial cell migration. GO analysis of cellular component terms revealed that *MOXD1* is involved mainly in the following processes: cell–substrate junction; basement film; ER lumen; and cell–cell junction. GO analysis of molecular function terms revealed that *MOXD1* was involved mainly in growth factor binding, fibronectin binding, and transforming growth factor beta binding (Figure 6a) (Additional file 1). KEGG analysis revealed that *MOXD1* was involved mainly in ECM receptor interactions, the PI3K/Akt signaling pathway, the MAPK signaling pathway, and the cGMP/PKG signaling pathway (Figure 6b) (Additional file 1). Our PPI network analysis revealed that the *MOXD1* protein interacted with STX7, LUM, TAAR5, TAAR6, SLC18B1, TMEM163, PF4, SRXN1, CPLX3, COL1A2, and other proteins (Figure 6c). Finally, GSEA revealed that *MOXD1* was associated with EMT, angiogenesis, the interleukin (IL)-6/JAK/STAT3 signaling pathway, the inflammatory response, and hypoxia (Figure 6d) (Additional file 1).

### 3.5 Molecular characterization and chemosensitivity of *MOXD1* mRNA expression

**Figure 7:**
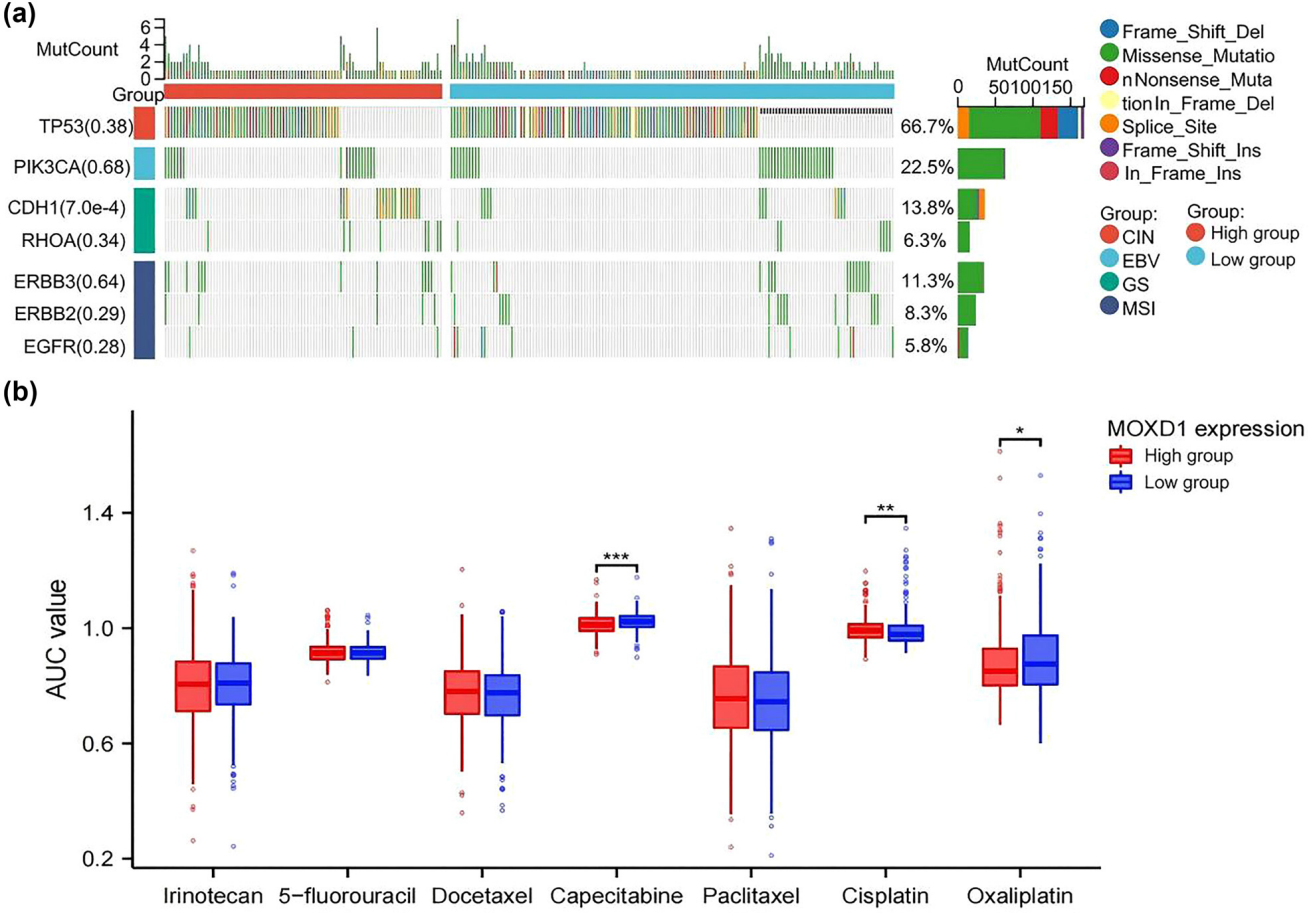
(a) Association between *MOXD1* mRNA expression levels and molecular classification in the TCGA-STAD cohort. (b) Association between *MOXD1* mRNA expression levels and chemotherapy sensitivity in the training cohort.

Considering the molecular classification standards of GC, correlations between mRNA *MOXD1* and four categories of GC in TCGA-STAD were analyzed. The results indicate that patients with high *MOXD1* mRNA expression were more likely to have CDH1 mutations (Figure 7a). The difference in chemosensitivity between the high- and low-*MOXD1* mRNA expression groups was also analyzed. Although patients with low *MOXD1* mRNA expression were more likely to respond to cisplatin, patients with high *MOXD1* mRNA expression were more likely to respond to capecitabine and oxaliplatin (Figure 7b).

### 3.6 Clinical significance of the MOXD1 protein

**Figure 8:**
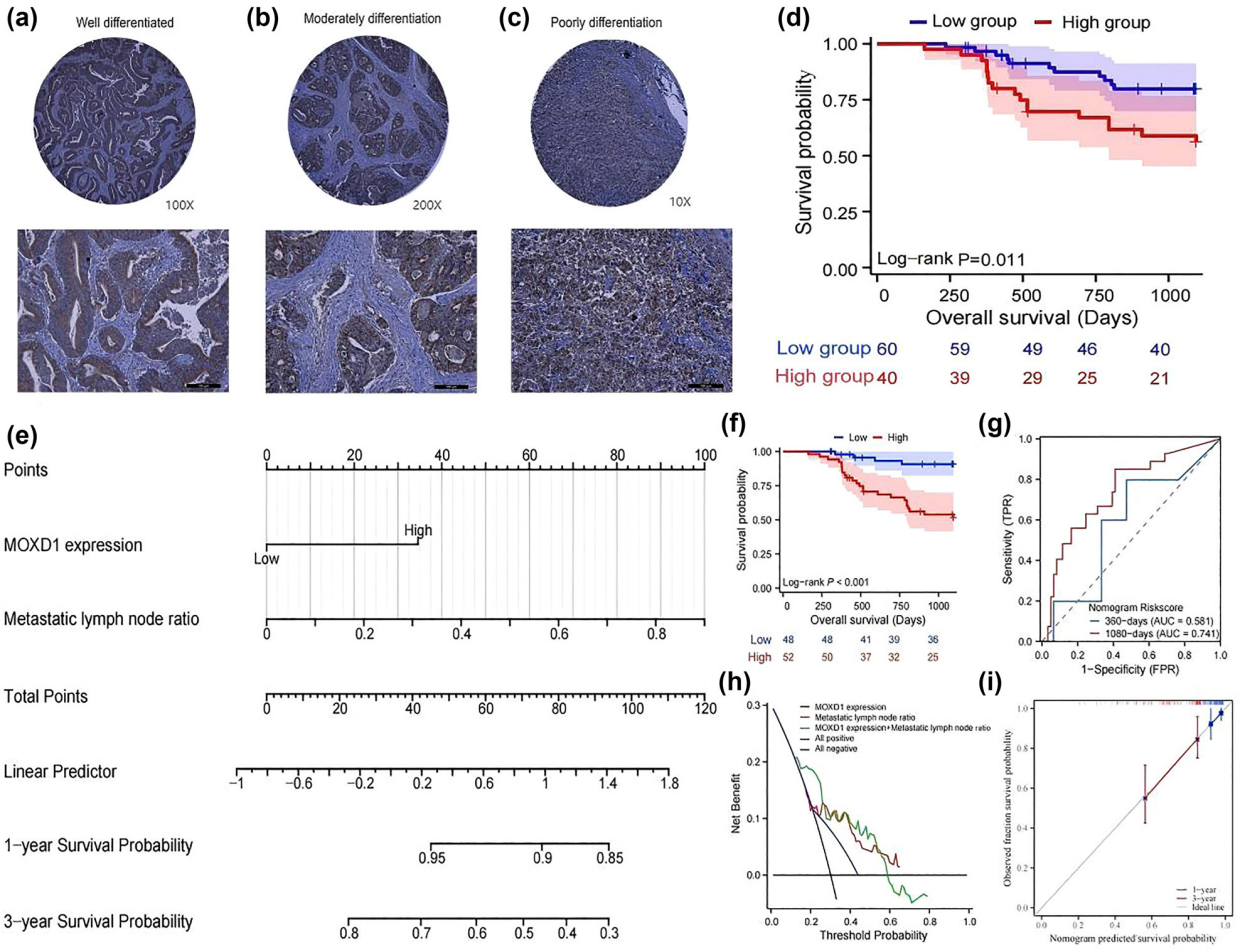
(a)–(c) IHC staining of TMAs for MOXD1 (well-differentiated, moderately differentiated, and poorly differentiated). (d) K‒M survival curve for MOXD1 expression in TMAs. (e) Nomogram constructed using MOXD1 expression and the metastatic lymph node ratio in TMAs. (f) K‒M survival curve for the nomogram. (g) timeROC curve for the nomogram. (h) DCA curve for the nomogram. (i) Calibration curve for the nomogram.

The immunohistochemical results for the TMAs are shown in Figure 8(a)–(c). The expression of *MOXD1* is not related to histological type. The survival curve indicated that patients with high *MOXD1* protein expression had poor survival (HR, 2.576; 95% CI, 1.199–5.537; *P* = 0.011) (Figure 8d). Cox regression analysis revealed that *MOXD1* protein expression and the metastatic lymph node ratio were independent risk factors related to prognosis (*P* = 0.020 and *P* = 0.047, respectively) (Table 3). Chi-square analysis revealed that *MOXD1* protein expression was closely related to N stage, pTNM stage, and the metastatic lymph node ratio (all *P* < 0.001) (Table 4). Next, the independent prognostic factors were combined to construct a nomogram (Figure 8e). According to the nomogram, the survival time of patients in the high-risk group was significantly shorter (HR: 6.533; 95% CI: 3.112–13.713; *P* < 0.001) (Figure 8f). The areas under the curve for evaluating 1- and 3-year survival were 0.581 (0.336–0.826) and 0.741 (0.629–0.854), respectively (Figure 8g). DCA and calibration curve analysis revealed that the nomogram exhibited good potential for evaluating patient prognosis, with a C-index of 0.711 (0.661–0.761) (Figure 8h and i).

**Table 3:** Univariate and multivariate analyses of TMAs based on Cox regression

Characteristics	Total (*N*)	Univariate analysis		Multivariate analysis	
		Hazard ratio (95% CI)	*P* value	Hazard ratio (95% CI)	*P* value
**MOXD1 expression**	100				
Low	60	Reference		Reference	
High	40	2.590 (1.212–5.532)	**0.014**	2.608 (1.159–5.869)	**0.020**
**Sex**	100				
Male	72	Reference			
Female	28	0.851 (0.362–2.002)	0.712		
**Age (years)**	100	0.992 (0.957–1.028)	0.646		
**BMI (kg/m** ^**2**^)	100	0.944 (0.845–1.054)	0.303		
**Tumor infiltration pattern**	100				
INFb	16	Reference			
INFa	20	0.674 (0.168–2.695)	0.577		
INFc	48	1.186 (0.394–3.576)	0.761		
N/A	16	1.228 (0.330–4.573)	0.760		
**Lymphatic infiltration**	100				
Negative	55	Reference			
Positive	45	0.940 (0.445–1.988)	0.872		
**Venous infiltration**	100				
Negative	70	Reference			
Positive	30	0.592 (0.240–1.460)	0.255		
**Nerve infiltration**	100				
Negative	25	Reference			
Positive	75	2.243 (0.778–6.471)	0.135		
**Tumor location**	100				
Lower third	54	Reference		Reference	
Middle and Upper third	42	1.866 (0.847–4.113)	0.122	1.324 (0.577–3.040)	0.508
Entire stomach	4	7.426 (2.017–27.337)	**0.003**	3.750 (0.710–19.814)	0.120
**Histological type**	100				
Mucinous	8	Reference			
Well to moderately differentiated	46	3.095 (0.409–23.430)	0.274		
Poorly differentiated	26	1.832 (0.214–15.686)	0.581		
Signet ring cell	20	3.485 (0.428–28.348)	0.243		
**HER2 expression**	100				
Negative	82	Reference			
Positive	18	1.660 (0.705–3.906)	0.246		
**CEA**	100				
≤5 ng/mL	86	Reference			
>5 ng/mL	14	0.679 (0.205–2.250)	0.526		
**CA-199**	100				
≤37 U/mL	88	Reference			
>37 U/mL	12	1.745 (0.663–4.593)	0.260		
**pTNM stage**	100				
I	10	Reference			
II	32	2.155 (0.259–17.902)	0.477		
III	58	4.558 (0.613–33.907)	0.138		
Metastatic lymph node ratio	100	14.056 (3.348–59.004)	**<0.001**	6.767 (1.023–44.780)	**0.047**

BMI: body mass index; CEA: carcinoembryonic antigen; HER2: human epidermal growth factor receptor 2. The histological type and pTNM stage were determined according to the 8th AJCC system. Bold values represent the category headings.

**Table 4:** Relationships between the expression of MOXD1 and clinicopathological characteristics

Characteristics	Low MOXD1 expression	High MOXD1 expression	*P* value
n	103	77	
**Sex,** ***n*** **(%)**			0.815
Male	76 (42.2%)	58 (32.2%)	
Female	27 (15%)	19 (10.6%)	
**Age (years), mean ± SD**	59.913 ± 10.051	60.74 ± 8.6121	0.562
**BMI (kg/m** ^**2**^ **), median (IQR)**	23.14 (20.73, 25.43)	22.66 (20.58, 24.57)	0.455
**Tumor infiltration pattern,** ***n*** **(%)**			0.278
INFb	30 (16.7%)	14 (7.8%)	
INFa	17 (9.4%)	19 (10.6%)	
INFc	39 (21.7%)	29 (16.1%)	
N/A	17 (9.4%)	15 (8.3%)	
**Lymphatic infiltration,** ***n*** **(%)**			0.911
Negative	58 (32.2%)	44 (24.4%)	
Positive	45 (25%)	33 (18.3%)	
**Venous infiltration,** ***n*** **(%)**			0.238
Negative	79 (43.9%)	53 (29.4%)	
Positive	24 (13.3%)	24 (13.3%)	
**Nerve infiltration,** ***n*** **(%)**			0.159
Negative	31 (17.2%)	16 (8.9%)	
Positive	72 (40%)	61 (33.9%)	
**Tumor location,** ***n*** **(%)**			0.566
Lower third	59 (32.8%)	38 (21.1%)	
Middle and upper third	41 (22.8%)	36 (20%)	
Entire stomach	3 (1.7%)	3 (1.7%)	
**Histological type,** ***n*** **(%)**			0.579
Well to moderately differentiated	46 (25.6%)	35 (19.4%)	
Poorly differentiated	22 (12.2%)	22 (12.2%)	
Signet ring cell	23 (12.8%)	14 (7.8%)	
Mucinous	12 (6.7%)	6 (3.3%)	
**HER2 expression,** ***n*** **(%)**			0.150
Negative	92 (51.1%)	63 (35%)	
Positive	11 (6.1%)	14 (7.8%)	
**CEA,** ***n*** **(%)**			0.600
≤5 ng/mL	91 (50.6%)	66 (36.7%)	
>5 ng/mL	12 (6.7%)	11 (6.1%)	
**CA-199,** ***n*** **(%)**			0.234
≤37 U/mL	93 (51.7%)	65 (36.1%)	
>37 U/mL	10 (5.6%)	12 (6.7%)	
**T stage,** ***n*** **(%)**			0.178
T1	7 (3.9%)	3 (1.7%)	
T2	20 (11.1%)	7 (3.9%)	
T3	35 (19.4%)	33 (18.3%)	
T4	41 (22.8%)	34 (18.9%)	
**N stage,** ***n*** **(%)**			**<0.001**
N0	39 (21.7%)	11 (6.1%)	
N1	23 (12.8%)	13 (7.2%)	
N2	19 (10.6%)	22 (12.2%)	
N3	22 (12.2%)	31 (17.2%)	
**pTNM stage,** ***n*** **(%)**			**<0.001**
I	19 (10.6%)	4 (2.2%)	
II	41 (22.8%)	15 (8.3%)	
III	43 (23.9%)	58 (32.2%)	
**Metastatic lymph node ratio, median (IQR)**	0.056 (0, 0.156)	0.156 (0.061, 0.322)	**<0.001**

BMI: body mass index; CEA: carcinoembryonic antigen; HER2: Human epidermal growth factor receptor 2; IQR: interquartile range. The histological type and pTNM stage were determined according to the 8th AJCC system. Bold values represent the category headings.

### 3.7 *MOXD1* knockdown significantly inhibited the proliferation and invasion of GC cells

**Figure 9:**
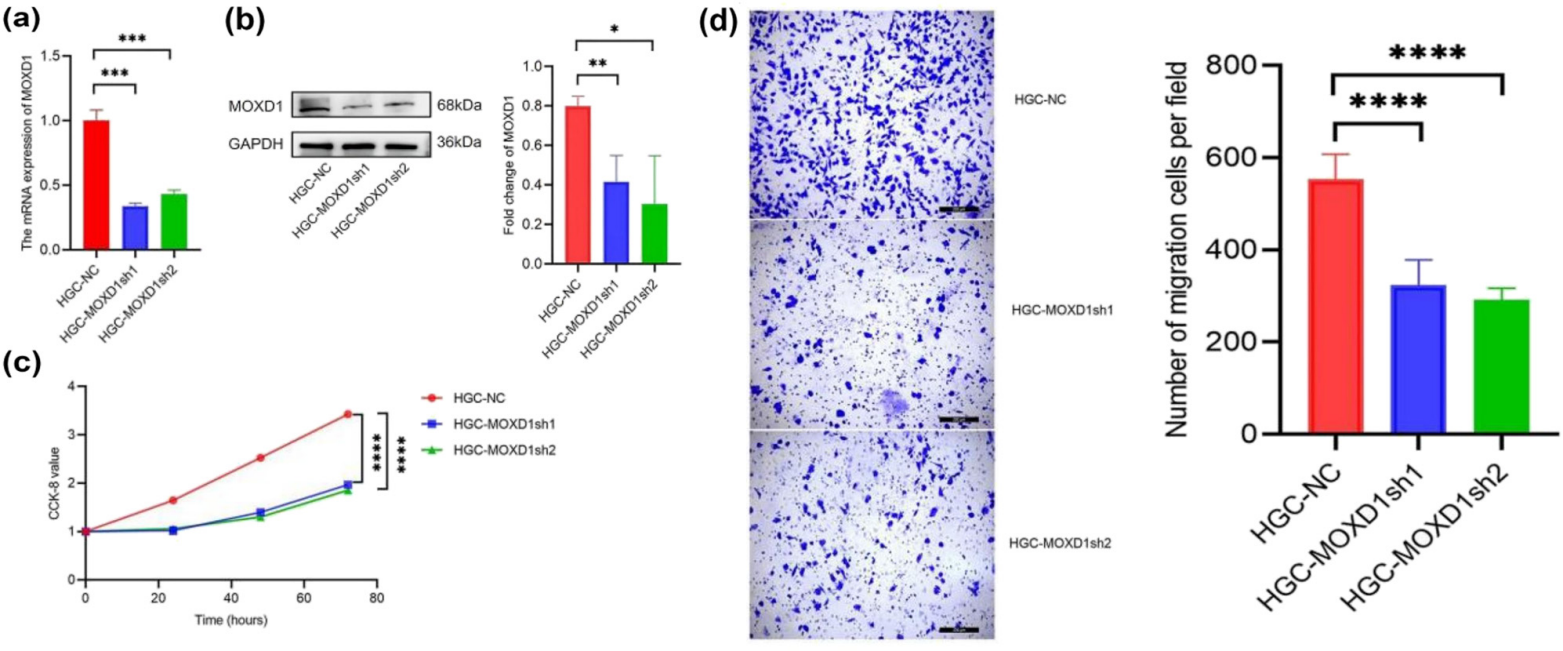
(a) qPCR analysis of HGC-NC, HGC-MOXD1sh1, and HGC-MOXD1sh2 cells. (b) Western blot analysis of HGC-NC, HGC-MOXD1sh1, and HGC-MOXD1sh2 cells. (c) CCK-8 assay results for HGC-NC, HGC-MOXD1sh1, and HGC-MOXD1sh2 cells. (d) Cell migration assay results for HGC-NC, HGC-MOXD1sh1, and HGC-MOXD1sh2 cells. All experiments were repeated at least three times.

Our qPCR and Western blot results provide strong evidence that *MOXD1* was markedly knocked down (at both the transcriptome and proteome levels) in HGC-27 cells infected with *MOXD1*-interfering viruses (Figure 9a and b). According to our CCK-8 assay results, the proliferation rates of HGC-27 cells after *MOXD1* knockdown (HGC-MOXD1sh1 group, HGC-MOXD1sh2 group) were significantly lower than those of control cells (HGC-NC group) (Figure 9c). Therefore, *MOXD1* protein knockdown affected the proliferation of GC cells. Moreover, our Transwell assay results revealed that the invasion capacities of HGC-MOXD1sh1 and HGC-MOXD1sh2 cells were significantly diminished (Figure 9d). Therefore, *MOXD1* protein knockdown also affected the invasion capacity of GC cells. Asterisk denotes statistically significant differences: *P < 0.05; **P < 0.01; ***P < 0.001; ****P < 0.0001.

## 4 Discussion

OS is an imbalance between oxidation reactions and antioxidation reactions in cells that results in excessive ROS production. ROS can originate from either internal sources, including mitochondria, neutrophils, and macrophages (by nicotinamide adenine dinucleotide phosphate oxidase), or from external sources, including ionizing radiation and external chemical stimulation. ROS generation may involve the production of 8-OH deoxyguanosine, which is involved in the transformation of GC pairs into TA pairs during DNA replication, resulting in chromosome instability, cell mutation, and potentially tumorigenesis. Although oxidative cleavage of RNA is more difficult than oxidative cleavage of DNA, 8-oxoguanosine, 8-hydroxyadenine, and other substances can affect the stability of RNA through base modification and excision, leading to abnormal protein translation processes. OS can also affect protein stability by altering amino acid chemical groups, including those of tyrosine and phenylalanine. Therefore, an overall reduction in ROS-induced mutations delays cancer development and is generally beneficial to patients [8,38,39].

In colorectal cancer patients, the score obtained from a 9 OS-related lncRNA construction could be used to predict prognosis [40]. IL (IL-6, IL-8, and IL-10) levels and tumor necrosis factor-α levels are highly correlated with the levels of malondialdehyde, a marker of OS, indicating that OS may be reflected by the immune response [41]. Additional processes, such as lipid metabolism and ferroptosis, are also closely related to OS [38,42]. Furthermore, OS is known to be associated with inflammation, neurodegenerative diseases, and cancer. In tumor cells treated with chemotherapy drugs, OS was also shown to affect the production of reactive species, ultimately modulating the therapeutic effect [43,44,45].

Although Wu et al. previously demonstrated the importance of OS in GC [11], GC is highly heterogeneous, necessitating elucidation of the role of OS in different GC patients. To identify potential molecules involved in OS, we first constructed an OS risk score related to prognosis using our training cohort. This method yielded two genes of interest, namely, *CYP1B1* and *APOD*. Cytochrome P450 family 1 subfamily B member 1 (CYP1B1) is a member of the cytochrome P450 family that participates in the metabolism of carcinogenic compounds, such as 7,12-dimethylbenz[a]anthracene, and plays a key role in estrogen metabolism [46]. Apolipoprotein D (APOD) is a member of the lipoprotein superfamily, and this transporter modulates lipid metabolism during several important BPs, including inflammatory and antioxidant reactions [47]. CYP1B1 and APOD have already been associated with tumor pathobiology in the literature, and their roles in various cancers are explored below.

CYP1B1 has been shown to play an important regulatory role in estrogen-related malignant tumors, such as breast, ovarian, and uterine cancers [46,48]. Although CYP1B1 is considered to have an inhibitory effect on many malignant tumors, HIF-1α can affect the expression of CYP1B1 by activating estrogen receptor α in breast cancer. CYP1B1 polymorphisms have been shown to modulate estrogen regulatory enzyme activity, promoting the occurrence of breast cancer [49,50]. Moreover, CYP1B1 polymorphisms are known predisposing factors in male patients with breast cancer. In bladder cancer, *CYP1B1* is associated with lymph node metastasis, and its expression increases with the progression of this disease [51]. In colorectal cancer cells, *CYP1B1* modulates the cell cycle by affecting the expression of the *PCNA* and *FEN1* genes, ultimately promoting proliferation [52]. As a potential therapeutic target, the knockdown of *CYP1B1* could increase the sensitivity of breast cancer cells to paclitaxel, 5-fluorouracil, and cisplatin. *CYP1B1* is also a potential target for anti-PD-1 treatment of colorectal cancer [53,54]. In GC, *CYP1B1* expression was observed to increase with tumor stage and grade, and it was highly correlated with tumor-related fibroblast numbers, immunological checkpoints, microsatellite instability, tumor mutation burden (TMB), and neoantigens. Moreover, CYP1B1 polymorphisms affect the progression of GC [55,56].

APOD is known to modulate important processes such as inflammation and antioxidation, and its expression is closely associated with numerous malignant tumors [47]. A risk model constructed using *APOD*, *APOC1*, and *SQLE* was used to evaluate the prognosis of patients with cervical cancer and their corresponding immune microenvironment status [57]. *APOD* was also found to be highly expressed in high-grade prostatic intraepithelial neoplasia and was identified as a potential biomarker [58]. In colorectal cancer, *APOD* was identified as a cancer stem cell-related gene [59]. *APOD* was also used as a potential marker for dexamethasone treatment of lymphoma [60]. In GC, *APOD* was identified as a gene affecting the TMB score and basement membrane, and high *APOD* expression was often associated with poor prognosis. Moreover, *APOD* was identified as a potential target for evaluating immunotherapy [61,62,63].

As discussed above, the biological functions of both *CYP1B1* and *APOD* are closely related to the OS process. Importantly, the OS risk score constructed with *CYP1B1* and *APOD* was an independent risk factor related to the prognosis of GC patients. Moreover, constructing an operational nomogram from the OS risk score, age, and pTNM stage was possible. This nomogram demonstrated good potential for exploring and evaluating GC prognoses. Together, the above results provide further evidence that OS is an important biological pathway affecting the progression of GC.

*MOXD1* encodes a member of the copper monooxygenase protein family (which includes dopamine β monooxygenase and the peptide glycine α hydroxylated monooxygenase), and it is involved in numerous biological functions, including copper ion binding and redox reactions. *MOXD1* is highly expressed in pituitary gland, salivary gland, and GBM cells, whereas *MOXD1* mRNA expression in gastric cells is a biomarker of early GC [28]. Lai et al. [64] reported that fat mass and the expression of obesity-associated protein (FTO), a demethylase, were positively correlated with *MOXD1* expression and that FTO affected both the methylation of m6A *MOXD1* mRNA and the prognosis of GC patients.

In the present study, we found that high *MOXD1* mRNA expression was positively correlated with the OS risk score and that *MOXD1* might also be involved in OS in patients with GC. GO analysis revealed that *MOXD1* can participate in the translation of a multifactor binding protein that binds to growth factor and fibronectin (among other proteins) and is involved in ECM organization. KEGG analysis revealed that *MOXD1* was associated with the PI3K/Akt signaling, MAPK signaling, and cGMP/PKG signaling pathways. Two of these pathways have previously been implicated in OS. For example, coptisine inhibits OS during the treatment of hyperuricemia by inhibiting the PI3K/Akt signaling pathway [65]. OS was also shown to be regulated by cryptotanshinone treatment of polycystic ovary syndrome via the MAPK/ERK signaling pathway [66]. Hence, KEGG analysis provides direct evidence that *MOXD1* activity is closely associated with these important signaling pathways and indirect evidence that *MOXD1* participates in the regulation of OS via these pathways. *MOXD1* is also considered a potential molecular target for multiple drug therapies.

In this study, the associations between *MOXD1* mRNA expression levels and treatment outcomes were further evaluated. Our results revealed that cisplatin therapy was more suitable for patients with low *MOXD1* mRNA expression levels, whereas capecitabine and oxaliplatin therapies were more suitable for patients with high *MOXD1* expression levels. Therefore, *MOXD1* mRNA expression levels provide guidance for clinical treatment. In addition, GSEA revealed associations between *MOXD1* and several important BP pathways, specifically hypoxia and angiogenesis. These BP pathways are closely related not only to OS but also to the progression of malignant tumors [67,68]. Although studies suggest that the occurrence of OS may affect EMT, further exploration is warranted to investigate the potential role of EMT in promoting GC progression by *MOXD1* [69]. According to the molecular typing of GC, an association also exists between high *MOXD1* expression levels in patients and the incidence of mutations in cadherin 1 (*CDH1*). *CDH1* mutations are typically hereditary, and *MOXD1* changes may also have a genetic basis [70].

The effects of *MOXD1* mRNA expression on the immune microenvironment were also evaluated. The ESTIMATE, stromal, and immune scores of patients with high *MOXD1* expression levels were higher than those of patients with low *MOXD1* mRNA expression levels. Indeed, the immune response in patients with high *MOXD1* mRNA expression levels was significantly greater than that in patients with low *MOXD1* mRNA expression levels, and these patients also presented lower tumor purity. Gong et al. [71] reported that low tumor purity was associated with poor prognosis, the EMT pathway, infiltration of specific immune cells (e.g., M2 macrophages), and immune cell inhibition by chemokines. In the present study, patients with high *MOXD1* mRNA expression levels were found to exhibit increased EMT and TIDE. Therefore, the effectiveness of immunotherapy should be evaluated in patients with low *MOXD1* expression levels.

ssGSEA and CIBERSORT analyses also revealed a close association between *MOXD1* and various immune cells. High mRNA expression of *MOXD1* was accompanied by high infiltration of M2 macrophages, activated NK cells, and resting dendritic cells. Conversely, low mRNA expression of *MOXD1* was accompanied by the infiltration of plasma cells, activated mast cells, and resting NK cells. The single-cell database provides evidence for links between *MOXD1* mRNA expression levels, plasma cells, and fibroblasts. In the tumor microenvironment, M2 macrophages are postulated to produce an immunosuppressive response that promotes the metastasis of cancer cells [72]. Zhu et al. reported that M2 macrophage infiltration in the tumor microenvironment of endometrial cancer was related to an increase in ROS. A similar relationship was reported for GBMs [15,73]. These results suggest that OS may cause M2 macrophage infiltration and that *MOXD1* mRNA expression reflects the degree of infiltration.

In addition, patients with high *MOXD1* mRNA expression exhibited high infiltration of NK cells, which are known to play a key role in tumor cell killing. Klopotowska et al. reported that the antitumor activity of NK cells was weakened by elevated OS levels [74], potentially leading to immune escape and the promotion of distant metastasis [75]. High OS levels may also promote an increase in tumor-related fibroblast numbers via a growth factor present in the tumor microenvironment, possibly fibroblast growth factor 2 or tumor growth factor β. Notably, *MOXD1* mRNA expression was also associated with fibroblasts in the GSE134520 dataset [76]. Finally, the observed differences in plasma cell infiltration may be due to different sequencing methods and patient populations.

To date, only a few studies on *MOXD1* expression in malignant tumors have been published. In bladder cancer [24], *MOXD1* was found to be associated with copper metabolism. In HGSOC [25], bioinformatics analysis revealed that *MOXD1* was a potential biomarker related to prognosis. In GBM [27], *MOXD1* knockdown inhibited the proliferation, migration, and invasion of GBM cells and triggered apoptosis in ER-associated mitochondria, ultimately impacting tumor progression. In the present study, *MOXD1* mRNA expression levels in tumor tissue samples from the training cohort were significantly increased (compared with those in the corresponding para-tumorous normal tissue samples). Additionally, patients with high *MOXD1* expression levels presented shorter survival times, a result that was verified in the validation cohort. Moreover, high *MOXD1* expression was found to promote lymph node metastasis in GC patients.

*MOXD1* mRNA expression levels could also be used for the prognostic grouping of patients with N0, N1-2, and stages II and III of GC. Specifically, the *MOXD1* expression levels in T3, N2, N3, and stage III GC patients were greater than those in the other groups. Therefore, *MOXD1* expression (at the transcriptome level) increases with tumor progression and metastasis. Moreover, *MOXD1* (at the protein level) should be considered a biomarker for, and may be involved in, the promotion of GC progression. A chi-square analysis of our immunohistochemistry results for TMAs revealed that *MOXD1* expression was associated with N stage and pTNM stage. Moreover, the survival of patients with high *MOXD1* expression was relatively short. High *MOXD1* expression levels are associated with later N stages, higher metastatic lymph node ratios, and later pTNM stages; therefore, the *MOXD1* protein is postulated to promote the progression of GC.

For clinical applications, we constructed a nomogram of *MOXD1* expression levels and metastatic lymph node ratios, two independent risk factors for prognosis in patients with GC. In evaluating the prognosis of patients, the C-index of the nomogram was 0.711. The clinical application potential of this nomogram was further confirmed via ROC analysis, DCA, and K‒M survival curve analysis. Although size and time limitations may constrain the applicability of this pilot study, our results could easily be expanded by increasing both patient numbers and follow-up times in a future study. To further validate the proposed function of *MOXD1*, we constructed a stable knockdown cell line in HGC-27GC cells, which is different from the cell line used in the study of Lai et al. [61]. Our results revealed that the deletion of *MOXD1* significantly inhibited the proliferation and invasion capabilities of HGC-27 cells. These results provide further confirmation of the biological function of *MOXD1* and validation of its use as a biomarker of GC.

Several shortcomings were identified in this study. First, this was a single-center retrospective study with a limited patient number and a limited follow-up time. Although *MOXD1* demonstrated good prognostic ability in our study, its clinical significance should be verified in a subsequent study involving more patients, more centers, and a longer follow-up. Second, the mechanisms by which *MOXD1* affects OS are not known and should be elucidated by *in vitro* and *in vivo* functional experiments.

## 5 Conclusions

The OS risk score is an independent prognostic factor in GC, and GC patients with a high score have a poor prognosis. *MOXD1* mRNA expression levels were positively correlated with the OS risk score, and GC patients with high *MOXD1* expression (at both the mRNA and protein levels) had shorter survival times. *MOXD1* knockdown inhibited both proliferation and invasion in HGC-27 GC cells. *MOXD1* is a potential prognostic biomarker related to OS in patients with GC.

## Abbreviations

APOD: Apolipoprotein D
AUC: Area under the receiver operating characteristic curve
BMI: Body mass index
BPs: Biological processes
CCK-8: Cell counting kit-8
CDH1: Cadherin 1
CEA: Carcinoembryonic antigen
CI: Confidence interval
CYP1B1: Cytochrome P450 family 1 subfamily B member 1
DAB: Diaminobenzidine
DCA: Decision curve analysis
DEGs: Differentially expressed genes
EMT: Epithelial–mesenchymal transition
ER: Endoplasmic reticulum
FDR: False discovery rate
FTO: Fat mass and obesity-associated protein
GBM: Glioblastoma
GC: Gastric cancer
GEO: Gene Expression Omnibus
GO: Gene Ontology
GSEA: Gene set enrichment analysis
HER2: Human epidermal growth factor receptor 2
HGSOC: High-grade serous ovarian cancer
HMU: Harbin Medical University
HR: Hazard ratio
IHC: Immunohistochemistry
IL: Interleukin
INFa: Expanding growth and a distinct border with the surrounding tissue
INFc: Infiltrating growth and an indistinct border with the surrounding tissue
INFb: In-between INFa and INFc
IQR: Interquartile range
KEGG: Kyoto Encyclopedia of Genes and Genomes
K‒M: Kaplan‒Meier
MOXD1: Monooxygenase DBH like 1
MSI: Microsatellite instability
OS: Oxidative stress
OSRGs: OS-related genes
PD-1: Programmed cell death protein 1
PPI: Protein‒protein interaction
ROS: Reactive oxygen species
STAD: Stomach adenocarcinoma
STRING: Search tool for retrieving interacting genes
TCGA: The Cancer Genome Atlas
TIDE: Tumor immune dysfunction and exclusion
timeROC: Time-dependent receiver operating characteristic
TMAs: Tissue microarrays
TMB: Tumor mutation burden
TNM: Tumor node metastasis

## Supplementary Material

Supplementary material
