# Supplementary material for: The mRNA MOXD1: Link to oxidative stress and prognostic significance in gastric cancer
Source: Open Med (Wars). 2025 Sep 19;20(1):20251271. doi: 10.1515/med-2025-1271 (PMC12452077; doi:10.1515/med-2025-1271)
Supplement: Supplementary material [file med-2025-1271-sm.pdf]

Supplementary material

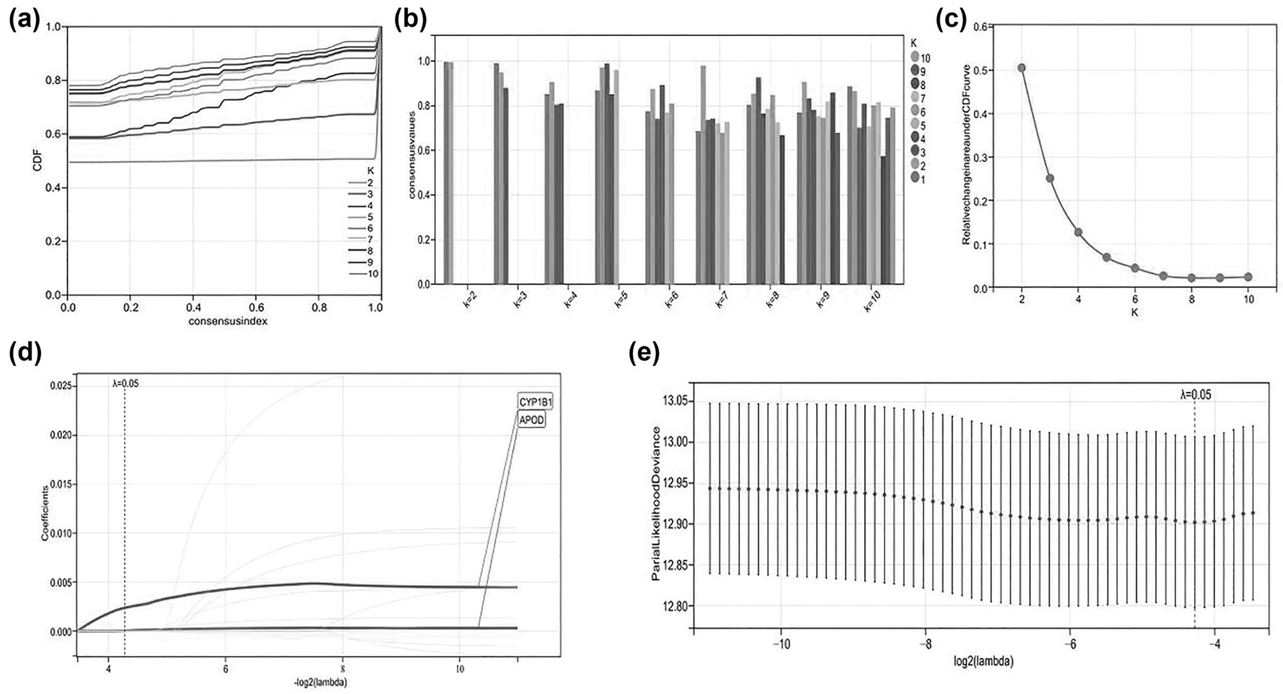

Figure S1: (a) The cumulative distribution curve of consensus. (b) The area under the curve of consensus. (c) Sample clustering consistency of consensus. (d) The result of Lasso-Cox analysis. (e) The lambda value of Lasso-Cox analysis.

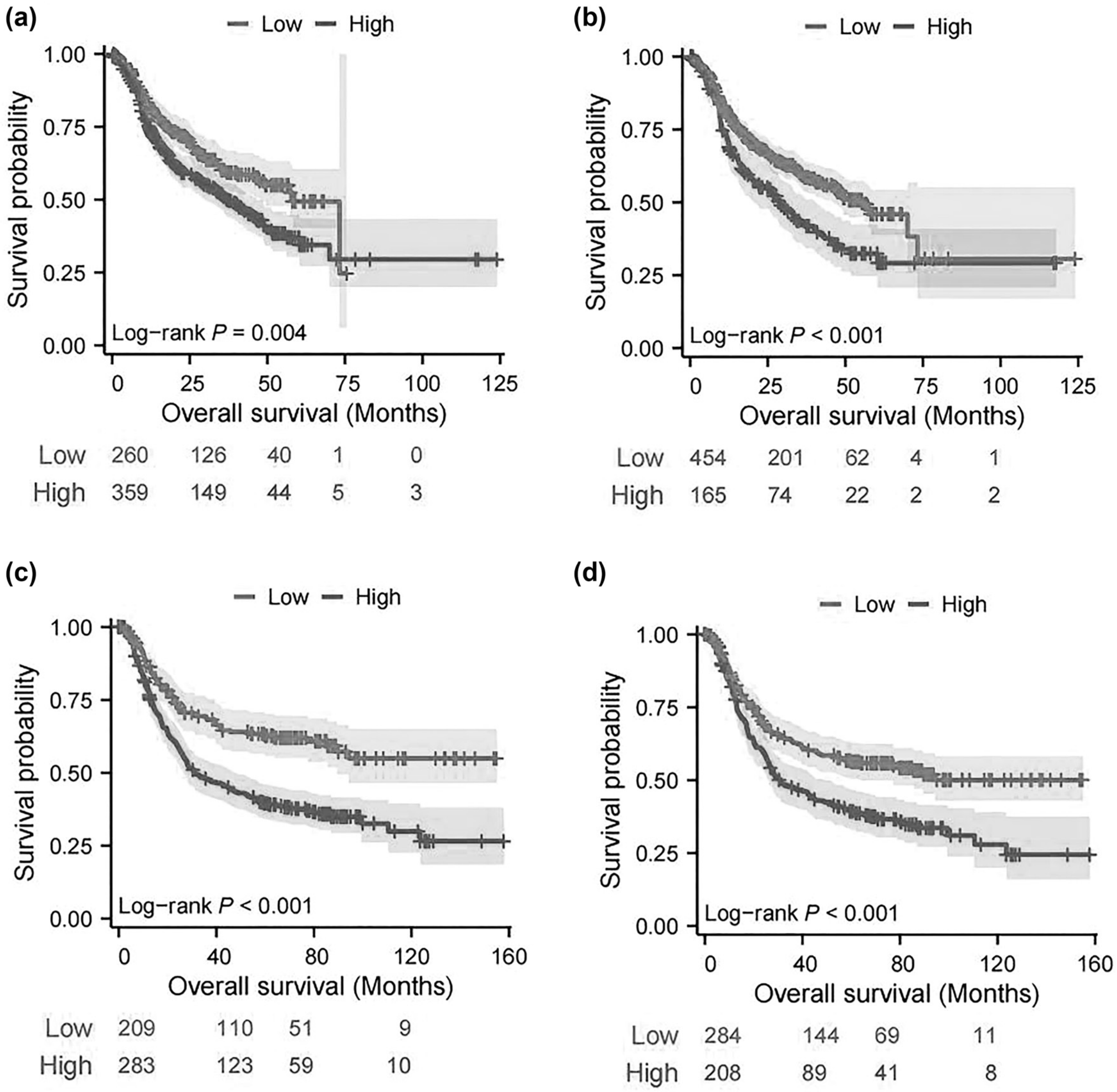

Figure S2: (a) and (b) Kaplan-Meier survival curve for APOD expression in the training cohort and validation cohort. (c) and (d) Kaplan-Meier survival curve for CYP1B1 expression in the training cohort and validation cohort.

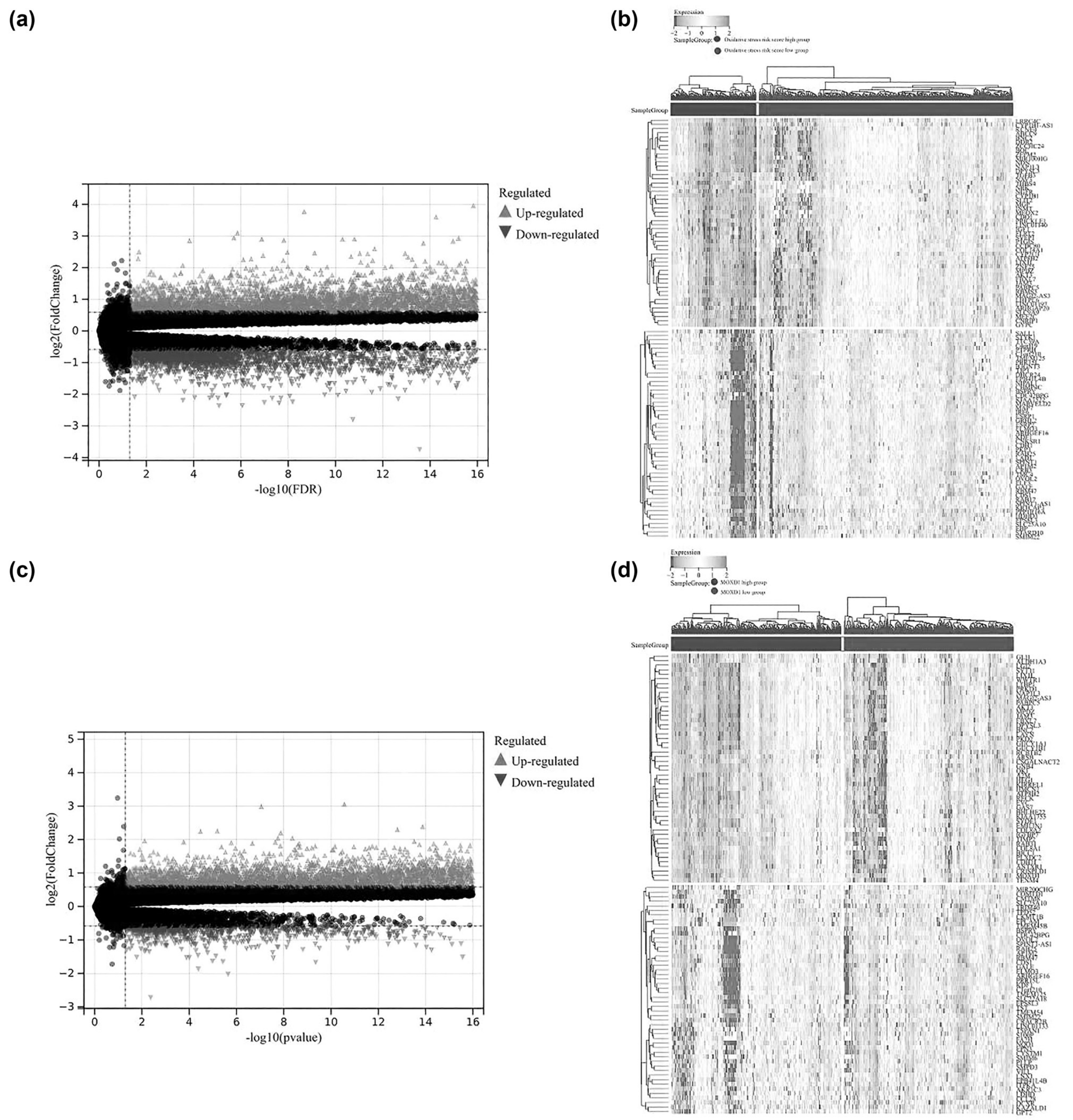

Figure S3: (a) and (b) Volcano plot and heatmap of differential gene expression between the high OS risk score group and the low OS risk score group. (c) and (d) Volcano plot and heatmap of differential gene expression between high MOXD1 expression and low MOXD1 expression groups.

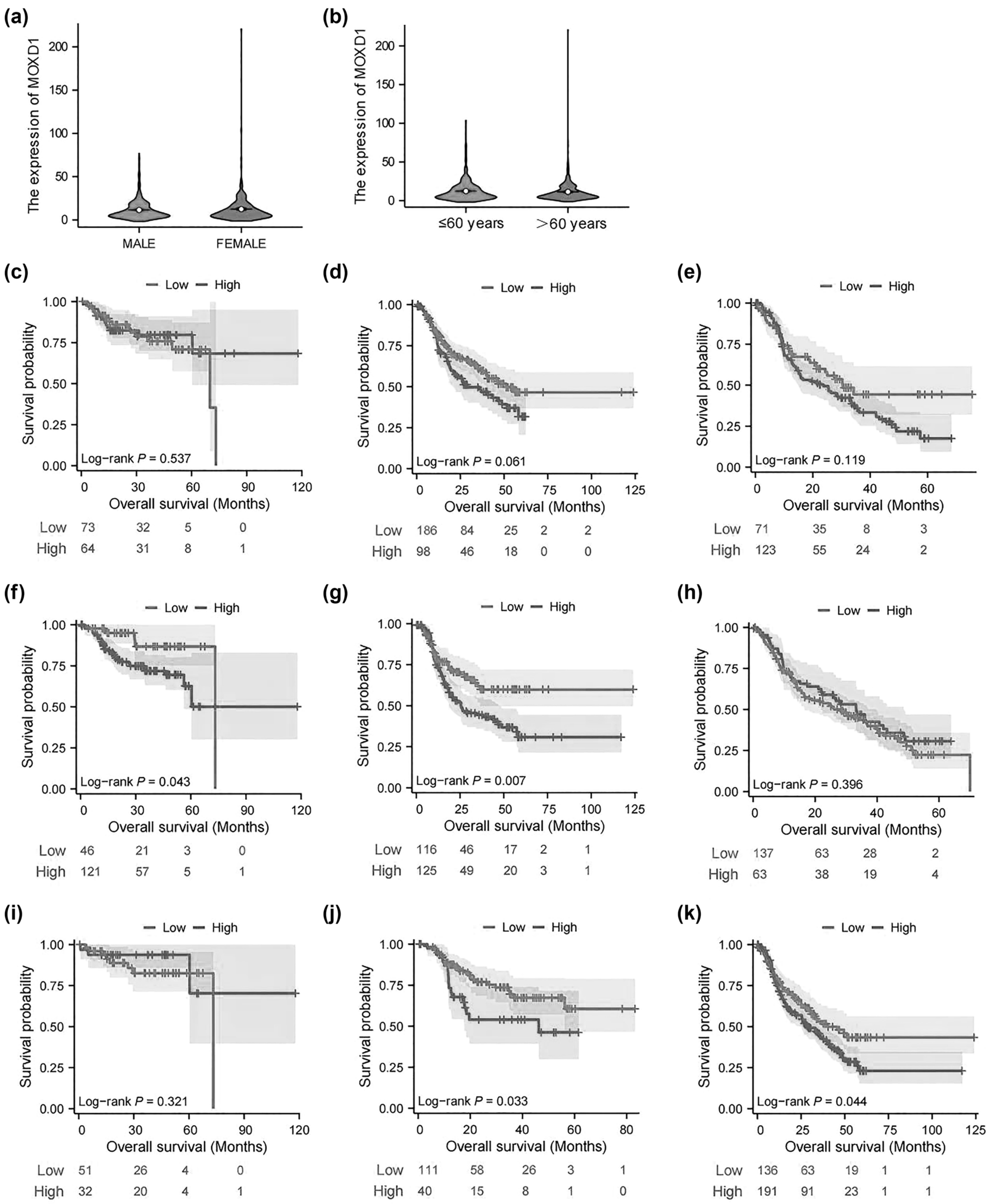

Figure S4: (a) and (b) Differential expression of MOXD1 between sex and age. (c)–(e) Kaplan-Meier survival curve of patients at T1-2 stage, T3 stage, and T4 stage based on MOXD1 expression. (f)–(h) Kaplan-Meier survival curve of patients at N0 stage, N1-2 stage, and N3 stage based on MOXD1 expression. (i)–(k) Kaplan-Meier survival curve of patients at stage I, stage II, and stage III based on MOXD1 expression.

Table S1: shRNA Sequences for MOXD1 Gene Knockdown

The shRNA of MOXD1 are CCGCATTATGTGCTCCTAGAA and GCGGTTATTGAATCCTGAGAA

Table S2: Histopathological data for Cohort 1 of 70 tumors from Australia

Expt number	Age	Gender	Lauren	Differentiation	Stage	Margins
GCa0001	73	M	Intestinal	Moderate	3a	R0
GCa0002	70	M	Intestinal	Moderate	3a	R0
GCa0006	85	F	Intestinal	Poor	3b	R0
GCa0008	61	M	Diffuse	Undifferentiated	3a	R0
GCa0010	68	F	Diffuse	Undifferentiated	2	R0
GCa0012	83	F	Diffuse	Poor	1b	R0
GCa0014	64	F	Intestinal	Well	2	R0
GCa0015	66	M	Intestinal	Poor	4	R0
GCa0016	78	M	Intestinal	Moderate	2	R0
GCa0018	65	M	Intestinal	Moderate	1a	R0
GCa0019	32	M	Intestinal	Poor	3b	R0
GCa0020	64	M	Diffuse	Poor	1b	R0
GCa0021	54	M	Diffuse	Undifferentiated	3a	R0
GCa0023	47	F	Diffuse	Undifferentiated	1b	R0
GCa0024	55	M	Intestinal	Well	3b	R0
GCa0026	61	F	Intestinal	Moderate	2	R1
GCa0028	70	F	Intestinal	Moderate	3a	R0
GCa0029	79	F	Intestinal	Poor	2	R0
GCa0031	60	M	Intestinal	Poor	4	R0
GCa0032	80	F	Diffuse	Undifferentiated	1b	R0
GCa0033	33	F	Diffuse	Poor	3a	R0
GCa0034	79	F	Mixed	Poor	3b	R0
GCa0036	81	M	Diffuse	Poor	3a	R0
GCa0037	76	F	Diffuse	Undifferentiated	3b	R0
GCa0039	75	M	Diffuse	Poor	3b	R0
GCa0041	55	M	Diffuse	Undifferentiated	3a	R0
GCa0043	57	M	Intestinal	Moderate	3a	R0
GCa0045	77	F	Intestinal	Poor	1b	R0
GCa0046	73	F	Intestinal	Moderate	2	R0
GCa0047	69	M	Intestinal	Moderate	3a	R0
GCa0049	74	M	Intestinal	Moderate	2	R0

(*Continued*)

Table S2: *Continued*

Expt number	Age	Gender	Lauren	Differentiation	Stage	Margins
GCa0050	67	M	Mixed	Poor	3b	R0
GCa0051	56	M	Mixed	Poor	4	R0
GCa0052	67	M	Intestinal	Moderate	2	R0
GCa0053	77	F	Intestinal	Moderate	2	R0
GCa0055	76	F	Diffuse	Undifferentiated	2	R1
GCa0057	79	M	Intestinal	Moderate	2	R0
GCa0058	73	M	Diffuse	Undifferentiated	4	R0
GCa0060	69	M	Intestinal	Moderate	2	R0
GCa0062	74	F	Diffuse	Undifferentiated	3b	R1
GCa0063	75	F	Diffuse	Undifferentiated	1b	R0
GCa0065	69	M	Diffuse	Poor	1b	R0
GCa0068	60	M	Intestinal	Moderate	3a	R0
GCa0070	36	M	Diffuse	Poor	3b	R0
GCa0072	59	F	Diffuse	Poor	1b	R0
GCa0073	70	M	Intestinal	Moderate	3a	R0
GCa0074	62	M	Diffuse	Poor	3b	R0
GCa0079	57	M	Intestinal	Poor	1b	R0
GCa0080	74	M	Intestinal	Moderate	1a	R0
GCa0082	62	M	Diffuse	Undifferentiated	4	R0
GCa0083	82	M	Diffuse	Undifferentiated	3b	R0
GCa0086	67	F	Intestinal	Moderate	3a	R0
GCa0087	53	M	Intestinal	Moderate	1b	R0
GCa0089	57	M	Diffuse	Undifferentiated	3b	R1
GCa0091	83	M	Intestinal	Moderate	2	R0
GCa0093	47	M	Diffuse	Undifferentiated	3a	R0
GCa0095	66	M	Intestinal	Moderate	4	R0
GCa0096	50	M	Diffuse	Undifferentiated	3b	R0
GCa0097	81	F	Diffuse	Undifferentiated	3b	R0
GCa0099	80	M	Mixed	Undifferentiated	3a	R0
GCa0101	71	M	Diffuse	Undifferentiated	3b	R0
GCa0103	71	M	Mixed	Poor	3a	R0
GCa0105	54	M	Intestinal	Poor	1b	R0
GCa0109	44	M	Diffuse	Undifferentiated	3a	R0
GCa0111	50	F	Diffuse	Poor	4	R0
GCa0113	51	M	Intestinal	Poor	2	R0
GCa0114	65	M	Intestinal	Poor	2	R0
GCa0115	85	M	Diffuse	Poor	.	R0
GCa0116	56	M	Mixed	Poor	2	R0
GCa0117	49	M	Intestinal	Moderate	3a	R0

Table S3: Histopathological data for Cohort 2 of 200 tumors from Singapore

TR Number	Age	Gender	Type	Lauren	Differentiation	T stage	Ming	Nodes	Grade
980056	33	F	Adeno		Poor	T3		N0	
970005	68	M	Adeno	Intestinal	Mod	T3		N0	G2
970003	38	F	Adeno		Poor	T3		N1	
970001	51	F	Adeno		Poor	T3		N1	
980048	48	F	Adeno		Poor	T3		N0	G3
980029	75	M	Adeno	Diffuse	Poor	T2		N1	
980028	58	M	Adeno		Mod	T2		N0	
980025	80	M	Adeno		Poor	T3		N3	
980021	59	M	Adeno		Mod	T3		N2	
980011	74	M	Adeno		Mod	T3		N1	
970010	72	M	Adeno		Mod	T3		N1	
970017	57	F	Adeno		Poor	T3		N0	
970032	55	F	Adeno			T1		N0	
970024	80	M	Adeno		Mod	T3		N0	
980035	40	M	Adeno	Intestinal	Mod	T3		N2	
980002	58	F	Adeno		Poor	T2		N1	
980148	66	M	Adeno		Poor	T3		N1	
980156	73	F	Carcinoid		Poor				
980161	66	F	Adeno		Poor	T3		N1	
980184	74	F	Adeno		Mod	T3		N2	
980211	63	M	Adeno	Intestinal	Mod	T3		N2	
980252		F	Adeno		Poor	T3		N1	
980255	74	M	Adeno		Mod	T3		N1	
980251	52	F	Adeno	Mixed		T1		N0	
980269	68	M	Adeno	Diffuse	Poor	T3		N3	
980305	38	F	Adeno		Poor	T3		N3	
980307	23	M	Adeno		Mod	T3		N2	
980097	65	M	Adeno			T2		N1	
980095	48	F	Adeno		Poor	T3		N3	
980063	49	F	Adeno		Poor	T3		N3	
46404174	47	F	Adeno	Diffuse	Poor	T2a	Infiltrative	N0	G3
48982424	53	F	Adeno	Diffuse	Poor	T2b	Infiltrative	N0	G3
9432729	61	F	Adeno		Mod	T3		N2	
76629543	92	M	Adeno	Intestinal	Mod	T3	Infiltrative	N0	G2
6412929	73	F	Adeno	Diffuse	Poor	T3	Infiltrative	N0	G3
5218170	66	F	Adeno	Diffuse	Poor	T3	Infiltrative	N2	G3
66811693	68	M	Adeno	Intestinal	Mod	T1	Expansive	N1	G2
82973565	69	M	Adeno	Mixed	Poor	T3	Infiltrative	N1	G3
26109694	64	M	Adeno	Diffuse	Poor	T2a	Infiltrative	N0	G3
73291145	47	M	Adeno	Intestinal	Well	T2b	Infiltrative	N1	G1

(*Continued*)

Table S3: *Continued*

TR Number	Age	Gender	Type	Lauren	Differentiation	T stage	Ming	Nodes	Grade
61669256	56	M	Adeno	Diffuse	Mod	T3	Infiltrative	N2	G2
15295992	67	M	Adeno	Intestinal	Mod	T2a	Infiltrative	N0	G2
57689477	68	F	Adeno	Intestinal	Mod	T1	Infiltrative	N0	G2
87622942	56	M	Adeno	Diffuse		T3	Infiltrative	N2	
65256293	65	F	Adeno	Diffuse	Poor	T3	Infiltrative	N3	G3
96141474	75	M	Adeno	Intestinal	Mod	T2b	Infiltrative	N1	
91596276	68	F	Adeno	Mixed	Poor	T3	Infiltrative	N0	G3
21080055	53	M	Adeno	Intestinal	Poor	T2b	Infiltrative	N1	G3
74765340	72	F	Adeno	Intestinal	Mod	T2b	Infiltrative	N2	G2
49375233	79	F	Adeno	Intestinal	Mod	T3	Infiltrative	N3	G2
91228050	73	M	Adeno	Mixed		T4	Infiltrative	N2	
20263644	63	F	Adeno	Diffuse		T2b	Infiltrative	N1	
58947266	80	M	Adeno	Intestinal	Mod	T3	Infiltrative	N1	G2
32226415	77	M	Adeno	Intestinal	Poor	T2b	Expansive	N1	G3
38877042	65	M	Adeno	Diffuse	Poor	T3	Infiltrative	N1	G3
47492137	62	M	Adeno	Diffuse	Poor	T3	Infiltrative	N2	G3
31231321	71	M	Adeno	Diffuse		T3	Infiltrative	N3	
63540193	72	M	Adeno	Mixed	Mod	T2b	Infiltrative	N0	G2
75554796	78	F	Adeno	Intestinal	Mod	T3	Infiltrative	N1	G2
83507354	62	M	Adeno	Intestinal	Poor	T3	Infiltrative	N1	
2000484	68	M	Adeno	Intestinal	Mod	T3		N1	
2000521	67	F	Adeno		Poor	T2b		N2	
2000518	56	M	Adeno	Diffuse	Poor	T2		N0	
2000529	77	M	Adeno	Intestinal	Mod	T2		N1	
2000616	57	M	Adeno		Poor	T2b		N1	
2000617	77	M	Adeno		Poor	T2b		N2	
2000618	67	M	Adeno		Mod	T2a		N1	
2000619	69	F	Adeno		Poor	T3			
2000639	69	M	Adeno	Intestinal	Mod	T2b		N2	
2000676	85	F	Adeno		Mod	T3		N1	
2000696	80	F	Adeno		Poor	T2a		N0	
2000708	58	M	Adeno		Mod	T3		N1	
2000721	71	M	Adeno		Poor	T3		N2	
2000732	60	F	Adeno	Diffuse	Poor	T3		N1	G3
2000734	66	M	Adeno		Poor	T4		N1	
2000752	61	M	Adeno		Poor	T3		N2	
2000778	57	M	Adeno	Mixed	Poor	T3		N2	
2000859	65	M	Adeno		Poor	T3		N3	
2000877	45	M	Adeno		Poor	T2a		N1	
2000892	71	F	Adeno	Intestinal		T2		N1	

(*Continued*)

Table S3: *Continued*

TR Number	Age	Gender	Type	Lauren	Differentiation	T stage	Ming	Nodes	Grade
2000920	80	M	Adeno	Diffuse		T3		N2	
2000963	50	M	Adeno			T2			
2001086	80	M	Adeno	Diffuse	Poor	T3		N2	
2001120	67	M	Adeno		Mod	T3		N2	
2001123	56	M	Adeno		Mod	T3		N2	
2001135	64	M	Adeno		Poor	T3		N0	
2001159	78	M	Adeno	Intestinal	Well	T1		N0	
2001190			Adeno		Poor	T3		N2	
2001206	65	M	Adeno	Diffuse	Poor	T3		N2	G3
990413	68	M	Adeno		Poor	T3		N1	
990424	71	M	Adeno	Mixed	Mod	T2b		N1	
990475	71	M	Adeno		Well	T2a		N1	
990474	79	F	Adeno	Intestinal	Poor	T3		N0	
990489	79	M	Neuro			T2		N0	
990515	60	M	Adeno		Mod	T3		N2	
2000040	70	M	Adeno	Diffuse	Poor	T3		N2	
2000068	64	F	Adeno		Mod	T1		N0	
2000088	53	M	Adeno		Poor	T3		N1	
2000085	53	M	Adeno	Intestinal	Mod	T3		N0	
2000114	53	M	Adeno	Intestinal	Poor	T3		N2	G3
2000169	78	M	Adeno		Poor	T3		N2	
2000175	64	M	Adeno		Mod	T3		N1	
2000178	71	F	Adeno		Poor	T2b		N1	
2000201	70	M	Adeno	Intestinal	Well	T2b		N0	
2000238	74	F	Adeno	Diffuse	Poor	T3		N0	
2000242	67	F	Adeno	Intestinal	Mod	T2a		N0	
2000256			Adeno		Poor	T3		N1	
2000286	65	M	Adeno		Mod	T3		N0	
2000291	68	M	Adeno		Poor	T3		N1	
2000303	48	M	Adeno		Poor	T3		N1	
2000346	32	F	Adeno	Diffuse	Poor	T3		N1	
2000362	51	M	Adeno		Mod	T3		N2	
2000403	68	M	Adeno	Diffuse		T3		N1	
2000433	56	F	Adeno						
2000434	67	M	Adeno			T2		N0	
2000441	53	M	Adeno		Poor	T3		N2	
2000472	82	M	Adeno	Diffuse	Poor	T3		N1	
2000479	62	M	Adeno	Intestinal	Mod	T3		N2	
990170	41	F	Adeno		Poor	T3		N1	
990150	87	F	Adeno	Diffuse	Poor	T2		N1	

(*Continued*)

Table S3: *Continued*

TR Number	Age	Gender	Type	Lauren	Differentiation	T stage	Ming	Nodes	Grade
990136	76	M	Adeno		Mod	T1		N0	
990129	77	F	Adeno		Mod	T3		N1	
990119	56	M	Adeno		Poor	T3		N1	
990111	74	F	Adeno		Mod	T1		N0	
990108	65	M	Adeno	Intestinal	Mod	T2		N1	
990098	54	F	Adeno		Poor	T3		N2	
990097	46	M	Adeno	Intestinal	Poor	T3		N2	
990089	75	M	Adeno		Mod	T3		N2	
990090	64	M	Adeno	Intestinal	Mod	T3		N1	
990070	43	M	Adeno		Poor	T3		N1	
990071	71	F	Adeno		Mod	T3		N0	
990073	59	M	Adeno		Mod	T2		N2	
990069	69	M	Adeno	Diffuse	Poor	T2		N0	
990068	73	M	Adeno	Diffuse	Poor	T3		N1	
990060			Adeno	Diffuse	Poor	T3		N2	
990046	55	M	Adeno		Mod	T3		N2	
990044	69	M	Adeno		Mod	T3		N2	
990041	40	M	Adeno	Intestinal	Mod	T3		N1	
990024	68	M	Adeno		Mod	T3		N1	
990015	61	M	Adeno		Mod	T1		N0	
990010			Adeno		Poor	T3		N2	
990005	60	M	Adeno		Poor	T3		N2	
980447	69	M	Adeno		Poor	T3		N2	
980442	62	F	Adeno		Poor	T3		N1	
980437	68	F	Adeno		Poor	T3		N2	
980436	65	F	Adeno	Intestinal	Mod	T3		N1	
980418	88	M	Adeno		Mod	T3		N1	
980417	67	M	Adeno	Intestinal	Poor	T3		N2	
980401	83	F	Adeno	Diffuse	Poor	T3		N1	
980390	78	F	Adeno		Mod	T1		N1	
980386	76	F	Adeno	Diffuse	Poor	T3		N2	
980369	47	F	Adeno		Poor	T2		N0	
980344	70	F	Adeno		Poor			N1	
980327			Sq+Adeno		Poor	T3		N1	
980319	68	M	Adeno		Poor	T3		N1	
990172	60	M	Adeno	Intestinal	Mod	T2		N2	
990195			Adeno		Mod	T3		N1	
990203	79	M	Adeno		Poor	T3		N1	
990205	72	F	Adeno	Diffuse	Poor	T2		N1	
990228	40	F	Adeno		Poor	T3	Signet	N1	

(*Continued*)

Table S3: *Continued*

TR Number	Age	Gender	Type	Lauren	Differentiation	T stage	Ming	Nodes	Grade
990247	70	M	Adeno		Poor	T3		N0	
990275	72	M	Adeno	Intestinal	Well	T3		N0	
990300	50	F	Adeno		Poor	T3		N2	
990399			Adeno		Poor	T3		N2	
990355	34	F	Adeno	Diffuse	Poor	T3		N2	
990396	65	M	Adeno		Poor	T3		N2	
990412			Adeno		Poor	T3		N1	
57701999	88	M	Adeno	Intestinal	Mod	T3	Tubular	N1	G2
2001226	70	M	Adeno	Diffuse	Poor	T3	Signet	N2	
2001229	49	F	Adeno	Diffuse	Poor	T3	Signet	N0	
2001241			Adeno	Diffuse	Poor	T3	Signet	N0	
20020011	49	M	Adeno	Mixed	Poor	T3	Signet	N1	G3
20020032	56	M	Adeno			T2b			
20020195	68	M	Adeno		Poor	T3		N1	
20020361	63	M	Adeno		Poor	T2		N0	
20020448	64	M	Adeno		Poor	T2b		N2	G3
20020455	81	F	Adeno	Intestinal	Mod	T2		N0	
20020720	75	M	Adeno	Intestinal	Mod	T2a	Tubular	N1	G2
20020838	79	M	Adeno	Intestinal	Mod	T2b	Tubular	N0	
20020846	46	M	Adeno	Diffuse	Poor	T2b	Tubular	N1	G3
20020999	72	F	Adeno	Diffuse	Poor	T2a	Tubular	N2	G3
20021007	54	M	Adeno		Poor	T2b		N0	
20021146	71	F	Adeno	Diffuse	Mod	T1is	Tubular	N0	
10390127	27	F	Adeno	Diffuse	Poor	T3	Tubular	N0	G3
68334421	64	M	Adeno	Intestinal	Well	T2b	Papillary	N0	G1
69245824	41	F	Adeno	Diffuse	Poor	T3	Signet	N3	G4
47149013	77	M	Adeno	Mixed		T2b	Signet	N3	
98748381	66	M	Adeno	Mixed	Poor	T3	Signet	N2	G3
73813499	68	F	Adeno	Diffuse		T3	Signet	N2	
43658255	67	M	Adeno	Intestinal	Mod	T3	Tubular	N2	G2
37262942	88	F	Adeno	Diffuse	Poor	T2b	Tubular	N1	G3
78373410	51	F	Adeno	Diffuse		T3	Signet	N3	
31661621	84	F	Adeno	Diffuse	Mod	T2b		N2	G2
77263387	75	M	Large Cell Neuroendocrine			T2b		N1	
29806547	66	F	Adeno	Diffuse		T2b	Signet	N0	
91515473	43	M	Adeno	Mixed	Poor	T2b	Tubular	N2	G3
7847924	64	F	Adeno			T3		N1	

Table S4: Histopathological data for Cohort 3 of 31 tumors from the United Kingdom

RNA ID	Age	Gender	Lauren	Differentiation	Stage	Ming
3792	69.82	Male	Diffuse	Poor	3	Expansile
3793	67.24	Male	Intestinal	Moderate	3	Infiltrative
3794	76.39	Male	Diffuse	Poor	4	.
3798	56.85	Male	Intestinal	Poor	4	Infiltrative
3810	63.75	Male	Diffuse	Poor	1	Infiltrative
3811	83.73	Male	Intestinal	Moderate	2	Expansile
3812	75.6	Female	Diffuse	Poor	3	Infiltrative
3816	61.15	Male	Intestinal	Moderate	1	Expansile
3817	72.17	Male	Intestinal	Poor	4	Expansile
3820	71.41	Female	Intestinal	Well	1	Expansile
3857	55.21	Male	Mixed/Unclassifiable	Poor	3	Infiltrative
3860	77.7	Female	Intestinal	Moderate	3	Infiltrative
3861	73.31	Female	Diffuse	Moderate	4	Infiltrative
3862	56.16	Male	Intestinal	Moderate	1	Infiltrative
3863	53.04	Male	Mixed/Unclassifiable	Poor	3	Infiltrative
3864	74.74	Female	Intestinal	Moderate	3	Expansile
3865	77	Male	Intestinal	Moderate	2	Expansile
4352	74.57	Male	Intestinal	Poor	2	Infiltrative
3796	59.19	Female	Intestinal	Moderate	3	Expansile
3803	82.73	Female	Intestinal	Moderate	3	Expansile
3813	76.59	Female	Intestinal	Moderate	3	Expansile
3814	56.85	Male	Intestinal	Poor	4	Infiltrative
3815	70.9	Male	Intestinal	Poor	3	Infiltrative
3818	74.42	Male	Mixed/Unclassifiable	Poor	2	Infiltrative
3819	83.68	Female	Diffuse	Poor	3	Infiltrative
3858	78.78	Male	Intestinal	Well	1	Expansile
3859	78.55	Female	Intestinal	Moderate	3	Infiltrative
4349	70.08	Female	Intestinal	Moderate	3	Infiltrative
4350	83.09	Female	Intestinal	Poor	1	Expansile
4351	81.52	Female	Intestinal	Poor	3	Infiltrative

Table S5: The four molecular subtypes and patient characteristics

Characteristics	MSS/TP53-	MSS/TP53+	MSI	MSS/EMT	*P* value
N	107(35.7%)	79(26.3%)	68(22.7%)	46(15.3%)	
**Median age Sex**	65(30-82)	64(24-81)	66(31-84)	53(28-86)	0.0324a
Male	70(65.4%)	57(72.2%)	45(66.2%)	27(58.7%)	0.4863
Female	37(34.6%)	22(27.8%)	23(33.8%)	19(41.3%)	
**Location of tumor**					
Antrum	61(57.0%)	26(32.9%)	51(75.0%)	17(37.0%)	
Body	37(34.6%)	36(45.6%)	13(19.1%)	21(45.6%)	<0.0001
Cardia,GE junction	9(8.4%)	14(17.7%)	4(5.9%)	5(10.9%)	
Whole,multicentric	0(0.0%)	3(3.8%)	0(0.0%)	3(6.5%)	
**Grade and WHO classification**					
W/D and M/D tubular	54(50.5%)	32(40.5%)	33(48.5%)	4(8.7%)	
P/D tubular	37(34.6%)	31(39.2%)	29(42.7%)	19(41.3%)	<0.0001
Signet ring cell	9(8.4%)	5(6.3%)	3(4.4%)	20(43.5%)	
Mucinous	4(3.7%)	3(3.8%)	0(0.0%)	1(2.2%)	
Others	3(2.8%)	8(10.1%)	3(4.4%)	2(4.3%)	
**Lauren type**					
Intestinal	58(54.2%)	38(48.1%)	42(61.8%)	8(17.4%)	
Diffuse	42(39.3%)	36(45.6%)	20(29.4%)	37(80.4%)	<0.0001
Mixed	7(6.5%)	4(5.1%)	5(7.4%)	1(2.2%)	
Missing	0(0.0%)	1(1.2%)	1(1.0%)	0(0.0%)	
**pT stage**					
T2	70(65.4%)	57(72.1%)	47(69.1%)	14 (30.4%)	
T3	29(27.1%)	18(22.8%)	17(25.0%)	27(58.7%)	0.0003
T4	8(7.5%)	4(5.1%)	4(5.9%)	5(10.9%)	
**pN Stage**					
NO	12(11.2%)	4(5.1%)	16(23.5%)	6(13.0%)	
N1	41(38.3%)	45(57.0%)	31(45.6%)	14 (30.4%)	0.0058
N2	31(29.0%)	19(24.0%)	15(22.1%)	15(32.6%)	
N3	23(21.5%)	11(13.9%)	6(8.8%)	11(23.9%)	
**AJCC stage(6hed.)**					
Ib	10(9.4%)	4(5.1%)	14(20.6%)	2(4.3%)	
II	33(30.8%)	31(39.2%)	26(38.2%)	7(15.2%)	0.0011
III	33(30.8%)	26(32.9%)	19(28.0%)	18(39.1%)	
IV	31(29.0%)	18(22.8%)	9(13.2%)	19(41.3%)	
**EBV**					
Positive	2(1.9%)	12(15.2%)	0(0.0%)	4(8.7%)	0.0002
Negative	94(87.9%)	60(75.9%)	63(92.6%)	40(87.0%)	
Missing	11(10.2%)	7(8.9%)	5(7.4%)	2(4.3%)	
**Lymphovascular invasion**					

(*Continued*)

Table S5: *Continued*

**Characteristics**	**MSS/TP53-**	**MSS/TP53+**	**MSI**	**MSS/EMT**	***P* value**
Positive	76(71.0%)	55(69.6%)	43(63.2%)	31(67.4%)	0.3142
Negative	26(24.3%)	19(24.1%)	20(29.4%)	8(17.4%)	
Missing	5(4.7%)	5(6.3%)	5(7.4%)	7(15.2%)	
**Venous invasion**					
Positive	15(14.0%)	14(17.7%)	9(13.2%)	6(13.0%)	0.0757
Negative	38(35.5%)	37(46.8%)	38(55.9%)	16(34.8%)	
Missing	54(50.5%)	28(35.4%)	21(30.9%)	21(52.2%)	
**Perineural invasion**					
Positive	30(28.0%)	27(34.2%)	11(16.2%)	20(43.5%)	0.0013
Negative	56(52.3%)	43(54.4%)	47(69.1%)	13(28.3%)	
Missing	21(19.6%)	9(11.4%)	10(14.7%)	13(28.3%)	
Adjuvant chemotherapy±RT	50(46.7%)	46(58.2%)	29(42.6%)	19(41.3%)	0.1727

Abbreviations: W/D, well differentiated; M/D, moderately differentiated; P/D, poorly differentiated; EBV, Epstein Barr Virus; RT, radiotherapy; GE, Gastroesophageal; pT stage, pathological assessment of the primary tumor (pT); pN stage, pathological assessment of the regional lymph nodes (pN).
